# An Inducible and Reversible Mouse Genetic Rescue System

**DOI:** 10.1371/journal.pgen.1000069

**Published:** 2008-05-09

**Authors:** Hongkui Zeng, Kyoji Horie, Linda Madisen, Maria N. Pavlova, Galina Gragerova, Alex D. Rohde, Brian A. Schimpf, Yuqiong Liang, Ethan Ojala, Farah Kramer, Patricia Roth, Olga Slobodskaya, Io Dolka, Eileen A. Southon, Lino Tessarollo, Karin E. Bornfeldt, Alexander Gragerov, George N. Pavlakis, George A. Gaitanaris

**Affiliations:** 1 Omeros Corporation, Seattle, Washington, United States of America; 2 Human Retrovirus Section, Vaccine Branch, Center for Cancer Research, National Cancer Institute-Frederick, Frederick, Maryland, United States of America; 3 Department of Pathology, University of Washington, Seattle, Washington, United States of America; 4 Neural Development Section, Mouse Cancer Genetics Program, National Cancer Institute-Frederick, Frederick, Maryland, United States of America; Harvard Medical School, United States of America

## Abstract

Inducible and reversible regulation of gene expression is a powerful approach for uncovering gene function. We have established a general method to efficiently produce reversible and inducible gene knockout and rescue in mice. In this system, which we named iKO, the target gene can be turned on and off at will by treating the mice with doxycycline. This method combines two genetically modified mouse lines: a) a KO line with a tetracycline-dependent transactivator replacing the endogenous target gene, and b) a line with a tetracycline-inducible cDNA of the target gene inserted into a tightly regulated (TIGRE) genomic locus, which provides for low basal expression and high inducibility. Such a locus occurs infrequently in the genome and we have developed a method to easily introduce genes into the TIGRE site of mouse embryonic stem (ES) cells by recombinase-mediated insertion. Both KO and TIGRE lines have been engineered for high-throughput, large-scale and cost-effective production of iKO mice. As a proof of concept, we have created iKO mice in the apolipoprotein E (ApoE) gene, which allows for sensitive and quantitative phenotypic analyses. The results demonstrated reversible switching of ApoE transcription, plasma cholesterol levels, and atherosclerosis progression and regression. The iKO system shows stringent regulation and is a versatile genetic system that can easily incorporate other techniques and adapt to a wide range of applications.

## Author Summary

We describe a technology for the creation of inducible and reversible gene inactivation in mice. It combines two genetically modified mouse lines: a knock-out line with a tetracycline transactivator replacing the endogenous target gene, and a line in which a tetracycline-inducible cDNA of the target gene has been inserted into a specific genomic locus. A critical component of this system is the unique chromosomal loci we have identified and engineered that offer a platform for easy insertion of any gene of interest for tightly controlled expression. Because of its simple binary nature, allowing independent modification of each of the two components and possibility of use in a high-throughput mode, we believe that our system will be useful for multiple applications, such as introducing mutant or humanized form of the target gene as well as functional manipulating tools. We have applied this technology to the Apolipoprotein E (ApoE) gene and have demonstrated that: a) the expression of ApoE is strictly dependent on the presence of doxycycline, a tetracycline group antibiotic, in the mouse diet, b) in the absence of doxycycline (ApoE repressed) atherosclerotic plaques are formed, confirming the importance of ApoE in the process, and c) upon re-induction of ApoE in the animals with doxicyclin, atherosclerosis regressed.

## Introduction

In the post-genome era, a major challenge is deciphering the function of thousands of newly identified genes. One of the main approaches for studying gene function involves inactivation of genes in cells or animals using random (chemical or insertional) mutagenesis or gene targeting. A common problem with these methods stems from the fact that the gene of interest is usually mutated throughout the animal's life. As a result, 1) in many cases the mutation leads to embryonic or neonatal lethality, precluding the assessment of the gene's function in later life; 2) in viable mutants interpretation of observed phenotypes is often complicated by the inability to distinguish the direct effects of the gene loss at the time of observation from the results of developmental abnormalities caused by the gene loss earlier in life; 3) in still other cases, life-long absence of a gene product causes compensatory adjustments of activities of other genes precluding the elucidation of the function of the gene of interest. Conditional knockout and gene expression technologies, such as the Cre/lox-mediated tissue-specific knockout [1] and the tetracycline (Tet) regulated transcriptional activation system [2], can regulate gene expression in a more spatially and temporally controlled fashion. However, these technologies are often laborious to establish and the results are frequently variable.

Here we report the development of a system that provides for the inducible and reversible gene inactivation in the mouse and can also be readily scaled up for high-throughput applications. The iKO system is a binary approach based on the Tet-dependent regulatory technology. It involves the combination of two mouse lines – a KO line that expresses the Tet-transactivator (tTA or rtTA) in place of the gene of interest, and a TIGRE (for tightly regulated) line that contains the gene of interest under the control of the Tet-responsive element (TRE) at a predetermined genomic locus. It has the advantage of, 1) ability to turn genes on or off at will by adding or removing doxycycline (Dox) at any time during the animal's life, thus minimizing embryonic lethality, developmental effects, and compensatory effects; 2) high degree of regulation to any gene inserted at the TIGRE locus, which has been selected to confer minimal basal expression and high inducibility, and to insert any gene of interest in a single step by Cre/loxP recombination; 3) efficiency; the design allowing streamlined production of both KO and TIGRE mice makes it possible to generate iKOs for a large number of genes in a cost-effective manner; 4) flexibility; KO and TIGRE lines can be engineered independently and combined in numerous ways, making a wide range of applications possible.

As a proof of concept, we report the characterization of an iKO of the apolipoprotein E gene (ApoE iKO). ApoE plays a key role in regulating cholesterol metabolism and atherosclerosis progression. ApoE KO mice develop hypercholesterolemia and atherosclerosis that closely resemble the human conditions and are rapidly reversed when APOE protein is supplied [3],[4],[5],[6],[7],[8]. Thus, inducible and reversible regulation of ApoE expression could result in rapid physiological changes, which in turn can help assess the iKO technology. Furthermore, the phenotype of ApoE deficiency is quantifiable and very sensitive to leaky expression, allowing for the evaluation of the stringency of gene regulation by iKO technology [9]. Here we demonstrate that in the ApoE iKO mice, ApoE gene expression, as well as blood cholesterol levels, is tightly controlled by Dox. In the presence of Dox, ApoE is expressed and the cholesterol levels are low; in its absence, the reverse is observed. Furthermore, on examination of aortic atherosclerosis in the ApoE iKO mice we found that Dox treatment before the onset of atherosclerotic lesions completely prevented lesion formation and Dox treatment after extensive lesions had already formed resulted in regression of the lesions. These results demonstrate the reversibility of the iKO, leading to phenotype switching within the same animal. ApoE iKO is also useful in its own right as a novel model system for the study of molecular mechanisms underlying atherosclerosis progression and regression.

## Results

### Principle of the iKO System

**Figure 1:**
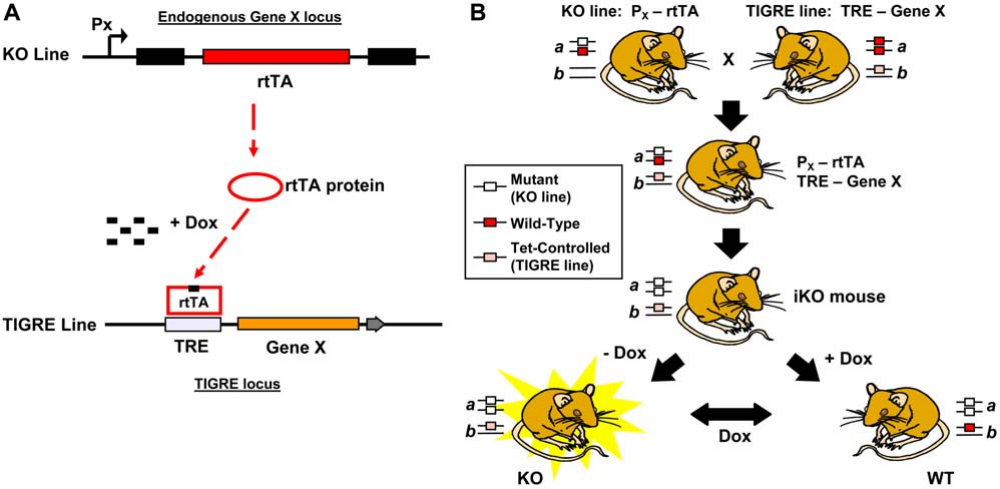
Schematic diagram of the principle of the iKO system. (A) Two components of the iKO system, a KO line and a TIGRE line. (B) Generation of iKO mouse by crossing the KO and TIGRE lines. The iKO is a composite mouse in which: 1) both copies of the endogenous Gene X are disrupted by the insertion; 2) rtTA is driven by the promoter of Gene X; and 3) the cDNA of Gene X is in the TIGRE locus under the control of TRE. Functional Gene X is only expressed from the TIGRE locus when rtTA is bound to Dox, and therefore is inducibly and reversibly regulated by Dox.

As illustrated in Figure 1A, two genetically modified mouse strains are created. The first is a KO line in which a Tet-dependent transactivator (rtTA in this example) is inserted into the target gene (Gene X). The insertion inactivates Gene X, and places rtTA under the control of the endogenous promoter of Gene X. The KO line can be generated via either homologous recombination or insertional mutagenesis. The second line (TIGRE) contains an additional copy of Gene X cDNA (or genomic fragments) driven by TRE-promoter inserted in a specific locus in the genome (the TIGRE locus), which has been pre-selected for low basal transcriptional activity and high inducibility. When these two lines are crossed, rtTA protein produced from the KO allele can activate the TRE-Gene X in the TIGRE locus only in the presence of Dox. (Alternatively, tTA can be used, which works in the opposite way – Tet-off instead of Tet-on.)

Figure 1B illustrates the breeding of KO and TIGRE lines to produce the iKO mouse, which is homozygous for the KO locus and carries one copy of the TIGRE allele. The status of the iKO mouse is regulated by Dox. In the absence of Dox, rtTA protein is produced, but is inactive. As a result, TRE-Gene X is silent, Gene X protein is not produced and the KO phenotype is manifested. In the presence of Dox, rtTA stimulates the synthesis of Gene X protein from the TIGRE locus in the same cells in which the endogenous gene would normally be expressed. Expression complements the missing endogenous Gene X activity and leads to phenotypically normal animals. Thus, one can switch between wild type and KO state of animals by simply adding or removing Dox (e.g. with food).

### Selection of TIGRE Loci

**Figure 2:**
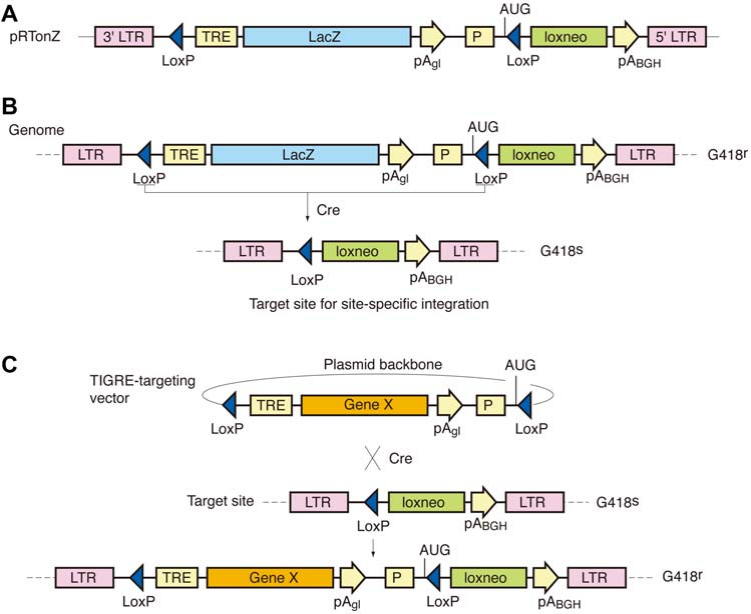
Retroviral vector used to search for tightly regulated loci and strategy to introduce a new gene into these predetermined loci. (A) Structure of the pRTonZ retroviral vector. To prevent effects of viral enhancer on the TRE promoter, the enhancer sequence was deleted in the 3′ long terminal repeat (LTR). Subsequent transduction into target cells is expected to lead to enhancer deletion in both LTRs. The insert was cloned in the opposite orientation of the LTRs, so that the polyA addition signals would not decrease the viral titer. (B) Excision of the lacZ reporter gene from TIGRE loci. LoxP-flanked sequences within the retroviral vector are removed by transient expression of Cre recombinase in ES cells, leaving a single copy of loxP site in the genome. ES cells become G418-sensitive since the loxneo gene loses its promoter and initiating AUG. (C) Introduction of a new gene into the TIGRE locus. The G418-sensitive ES cells selected in (B) are cotransfected with the TIGRE-targeting vector carrying a new gene (gene X) and the Cre expression vector. Proper Cre-mediated recombination between the TIGRE-targeting vector, containing the new gene, and the TIGRE site introduces the new gene into the TIGRE locus and converts the G418 sensitive ES cells into G418 resistant (the expected recombinant leads to neo expression by placing the PGK promoter and initiating AUG upstream of the loxneo gene). Symbols are: pA_gl_, rabbit β-globin gene polyA addition signal; P, mouse phosphoglycerate kinase-1 gene promoter; AUG, initiating AUG of neomycin phosphotransferase gene; loxneo, neomycin phosphotransferase gene having loxP sequence in-frame to initiating AUG; pA_BGH_, bovine growth hormone gene polyA addition signal; G418^r^, G418 resistant; G418^s^, G418 sensitive.

To screen for TIGRE loci, we constructed a Moloney murine leukemia virus (MoMLV)-based retroviral vector, pRTonZ (Figure 2A), in which the TRE-controlled lacZ gene was used as a reporter for gene regulation. Retroviral transduction at low multiplicity of infection ensures integration of a single copy of the TRE-lacZ unit into the genome. pRTonZ contains a modified neomycin phosphotransferase gene, loxneo, in which initiating AUG has been placed upstream of a loxP site in frame with the neo coding region. Once optimal locus is selected, it is utilized as a target site for transgene-integration by the scheme shown in Figures 2B and 2C. The TRE-lacZ unit is removed by Cre-mediated recombination of flanking loxP sites, leaving one loxP site and the neo gene in the genome (Figure 2B). Since the promoter of the loxneo gene as well as the initiating AUG are also removed, ES cells become G418-sensitive. In this configuration, any gene of interest can be introduced into the same locus by Cre/loxP recombination (Figure 2C). Recombinant ES clones can be selected by G418-resistance because the neo expression unit is reconstituted. PCR screening showed that >90% of these G418-resistant clones had correct insertion of the new gene.

**Figure 3:**
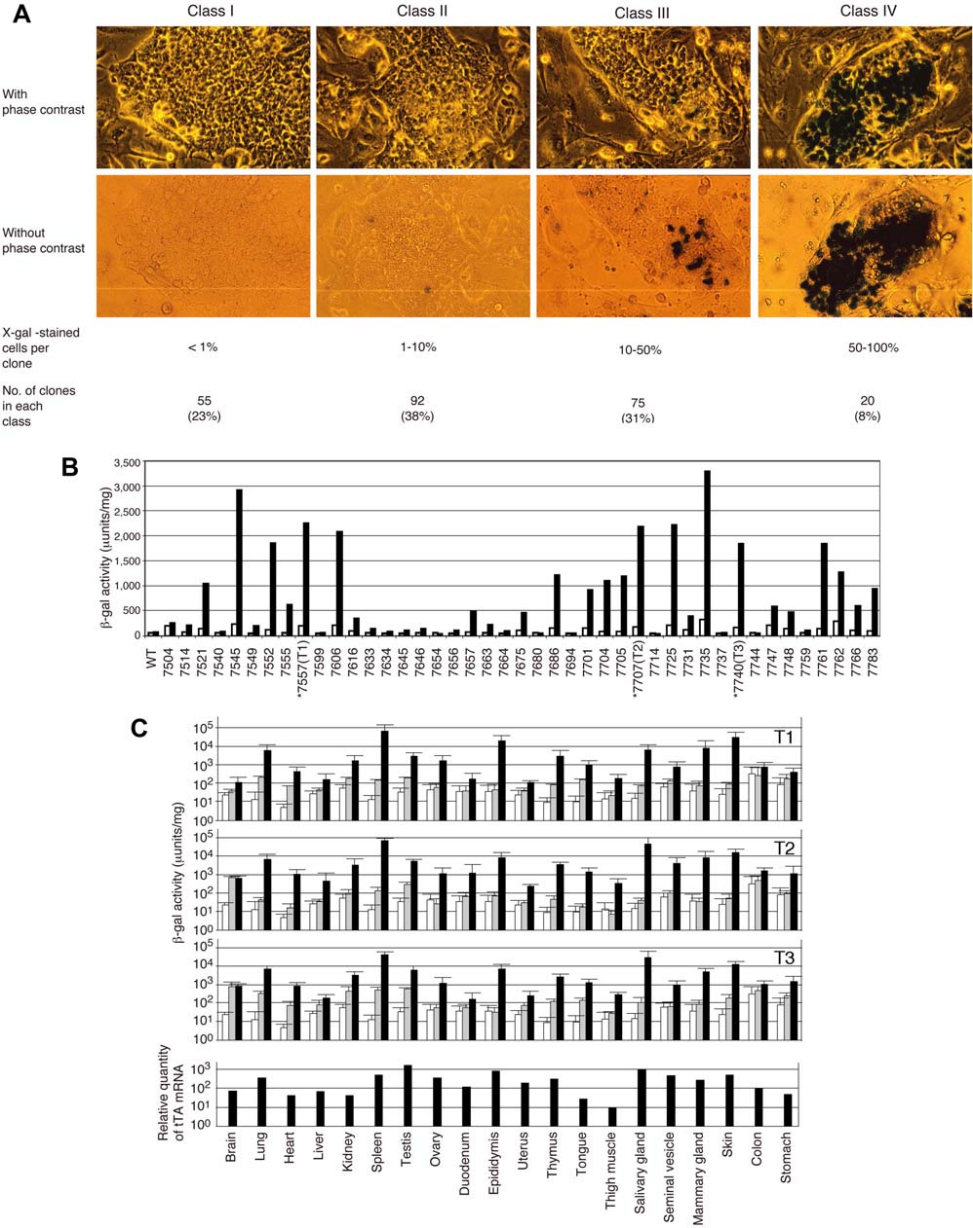
Characterization of gene regulation at TIGRE loci. (A) Screening for optimal integration sites in ES cells and classification of ES clones by X-gal staining. A representative ES clone is shown for each class with and without phase contrast for better imaging of ES cell morphology and X-gal staining, respectively. (B) Screening of class I clones for high inducibility. Forty three class I ES clones were transfected with a tTA expression vector and β-gal activity was quantified 48 hours post-transfection. Bars: open, without tTA; filled, with tTA. A luciferase expression vector was cotransfected to normalize β-gal activities. Three ES clones were designated as T1, T2 and T3 as shown in the figure, and were used to generate mice. (C) Gene regulation in mice generated from tightly regulated ES clones. Three mouse strains were established from ES clones T1, T2 and T3, and crossed to MMTV-tTA mouse. Top three panels: β-gal activity was measured in the following three genotypes: nontransgenic mice (lacZ(−)tTA(−), open bars); mice with lacZ gene but without tTA (lacZ(+)tTA(−), lightly shaded bars); mice with both lacZ gene and tTA gene (lacZ(+)tTA(+), filled bars). Values are shown as means with error bars of standard deviations from five male and five female animals. Values of nontransgenic mice (open bars) are common in all three panels. Bottom panel: tTA mRNA expression level quantified by real-time PCR from one male and one female mouse. Mean values are presented by filled bars. Note that values are shown in logarithmic scale.

Optimal loci were initially screened in ES cells. The ES cell line used was derived from CJ7 [10], of 129/Sv background. Following infection with the pRTonZ retroviral vector, G418-resistant (G418^r^) clones were stained with X-gal (Figure 3A). Most clones showed mosaic staining pattern. By the percentage of the X-gal-stained cell population, ES clones were classified into 4 categories (Figure 3A): class I (<1% of X-gal-stained cells) to class IV (>50% of X-gal-stained cells). From 242 ES clones analyzed, 55 clones were classified into class I. Inducibility was examined by β-galactosidase (β-gal) activity after transfecting 43 class I clones with a tTA expression vector (Figure 3B). We set the cut-off value for high induction level at 1500 µunits/mg of protein and nine clones belonged to this category.

Those loci were further examined in mice. Three independent TRE-lacZ mouse lines were generated from class I ES clones T1, T2 and T3 (Figure 3C). Heterozygous TRE-lacZ mice were crossed to MMTV-tTA mice, which has been reported to be transcriptionally active in a wide variety of cell types [11], allowing for the examination of lacZ induction in various tissues. Three genotypes of mice (lacZ(−)tTA(−), lacZ(+)tTA(−), and lacZ(+)tTA(+)) were analyzed for β-gal activity (Figure 3C). Activity of lacZ(−)tTA(−) represents endogenous eukaryotic β-gal activity. Difference between lacZ(−)tTA(−) and lacZ(+)tTA(−) indicates basal activity of lacZ gene in the absence of tTA, and comparison of lacZ(+)tTA(−) and lacZ(+)tTA(+) reveals induction levels in the presence of tTA. Overall, the three mouse lines showed similar pattern of β-gal activity, although some differences were also seen. Basal activities were low but detectable in many tissues, and they were comparable to the values measured in the parental ES clones. β-gal activity was inducible in almost every tissue, and overall induction levels correlated with the expression levels of tTA (Figure 3C, bottom panel).

### Improvement of Gene Regulation Control at the TIGRE Loci

**Figure 4:**
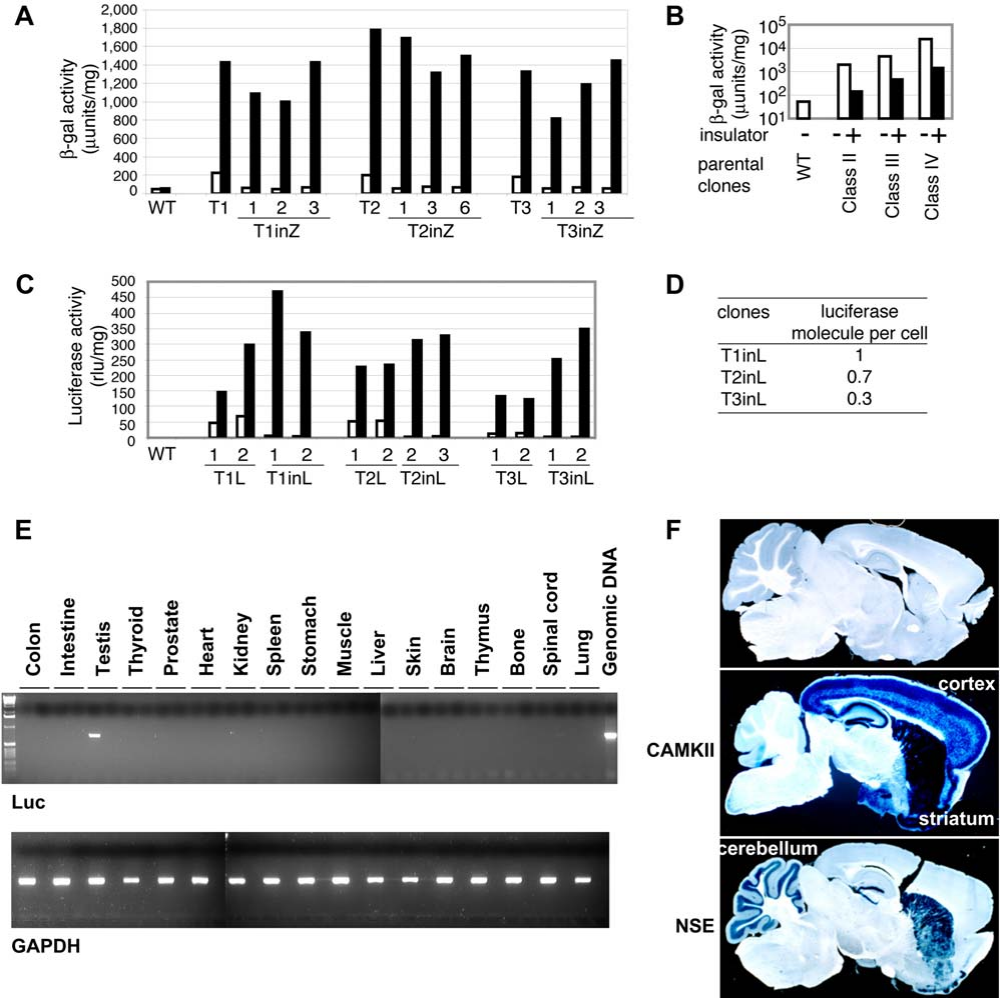
Tightening of gene regulation at TIGRE loci by insulators. (A) Insulator effect in class I clones. Parental ES clones without the insulator (T1, T2, T3) and clones with the insulator (T1inZ, T2inZ, T3inZ) were transfected by tTA expression vector, and β-gal activity was measured 48 hours post-transfection. Three independent integrant clones with insulator were analyzed in each integration site. A luciferase expression vector was cotransfected to normalize the β-gal activities. Clone numbers correspond to those in Figure S1. WT, wild type ES cells. Bars: open, without tTA; filled, with tTA. The same symbol was used in (B) and C). (B) Insulator effect on basal β-gal activity in class II, III and IV clones. Insulator sequence was introduced into three ES clones categorized in class II, III and IV of Figure 3A. Note that values are shown in logarithmic scale. (C) Insulator effect on the regulation of luciferase gene. The lacZ gene of the three class I clones (T1, T2, T3) were replaced by luciferase gene without (T1L, T2L, T3L) or with (T1inL, T2inL, T3inL) the insulator. Two independent integrants were established in each case. A lacZ expression vector was cotransfected to normalize the luciferase activities. Clone numbers correspond to those in Figure S2. (D) Number of luciferase molecule per cell in class I clones with the insulator sequence. (E) RT-PCR of the luciferase (Luc, upper panel) and positive control GAPDH (lower panel) transcripts in a TIGRE line (T1) with TRE-Luc and insulators. Each tissue has an RT-PCR reaction (RT+, left lane) and an RT- control (right lane) run simultaneously to exclude any possibility of genomic DNA contamination. The last lane is a positive control of genomic DNA PCR just for Luc. (F) β-gal staining of brain sagital sections (50 µm) of mice carrying a TRE-LacZ (+insulators) TIGRE (T1) line alone (top panel) or combined with either αCaMKII-tTA (middle panel) or NSE-tTA (bottom panel) transgene. There is no detectable β-gal staining in the absence of inducer (top panel). When TRE-LacZ TIGRE line is combined with αCaMKII-tTA, β-gal staining is seen in the same regions αCaMKII-tTA is expressed – mainly cortex, hippocampus and striatum (middle panel). When TRE-LacZ TIGRE line is combined with NSE-tTA, β-gal staining is seen in the same regions NSE-tTA is expressed – mainly striatum, dentate gyrus and cerebellum (bottom panel).

Although the three loci T1, T2 and T3 showed tight regulation of gene expression, basal activity was still detectable in many tissues. This could result from enhancers in the vicinity of the integration sites. To solve this problem, we flanked the TRE-lacZ reporter by the insulator sequence derived from the chicken β-globin locus [12],[13]. The insulator sequences were introduced into all three loci (T1, T2, T3) by Cre-mediated recombination according to the scheme shown in Figures 2B and 2C (see Figure S1 for details) and regulation of the lacZ gene was examined by transient expression of tTA in ES cells (Figure 4A). The insulators reduced basal β-gal activity to levels indistinguishable from wild type ES cells. In contrast, inducibility was not impaired by the insulator sequence, indicating its effectiveness for increasing stringency of gene regulation. The insulators were also introduced into class II, III and IV ES clones, which showed high basal activity (Figure 3A). Although the insulator sequences were effective, basal activity was still clearly detectable in every clone (Figure 4B). To test whether tight regulation is achieved by using other genes, we replaced the lacZ gene with a luciferase gene (Figure S2). With this reporter, insulators reduced basal activity by 14, 20 and 11 fold in T1, T2 and T3 loci, respectively, bringing it close to the instrument detection limit (Figure 4C). Calculation of the number of luciferase molecule per ES cell (Supporting Methods) revealed that on average only 1, 0.7 and 0.3 luciferase molecule were expressed per cell in T1, T2 and T3 loci, respectively (Figure 4D). Importantly, induced levels were not impaired by the insulator (Figure 4C), leading to high induction ratio of luciferase activity. Similar basal activity levels could also be achieved by expressing transrepressor (Figure S3). However, our system is simpler because no additional protein expression is required.

To evaluate the insulator effect in vivo, we also generated mice containing TRE-lacZ gene and insulators, or TRE-luciferase (Luc) gene and insulators at the TIGRE locus (T1), and bred them with mice containing tTA under the control of various promoters. Basal expression was examined in multiple tissues of TRE-Luc mice by RT-PCR (Figure 4E). Luc mRNA was undetectable in all tissues examined except testis, indicating very low basal levels of expression throughout the body. To examine if the Luc transcript detected in testis can produce functional Luc proteins, we conducted luciferase activity assays from protein extracts of all these tissues. The luciferase activity in testis was at the similar low basal level as all other tissues examined (data not shown) as well as the original TIGRE ES clone containing TRE-Luc with insulators that had been shown to express approximately one Luc molecule per cell (Figure 4D), suggesting that the RT-PCR band detected in testis resulted from aberrant transcription that did not generate functional protein. To examine induced expression, we used lines of mice with tTA under the control of two brain-specific promoters: α-CaMKII (calcium/calmodulin-dependent protein kinase II) [14] or NSE (neuron-specific enolase) [15]. Sagital sections of 50 µm thickness across the entire brain from TRE-lacZ, P_CAMKII_-tTA/TRE-lacZ or P_NSE_-tTA/TRE-lacZ mice were stained for β-galactosidase (gal) activity and representative sections are shown in Figure 4F. In TRE-lacZ mice, no β-gal staining was seen in any parts of the brain. In P_CAMKII_-tTA/TRE-lacZ or P_NSE_-tTA/TRE-lacZ mice, intense β-gal staining was observed in specific regions of the brain defined by the two promoters respectively. These results demonstrate tight control of the TIGRE locus in animals.

### Characterization of the TIGRE Locus

**Figure 5:**
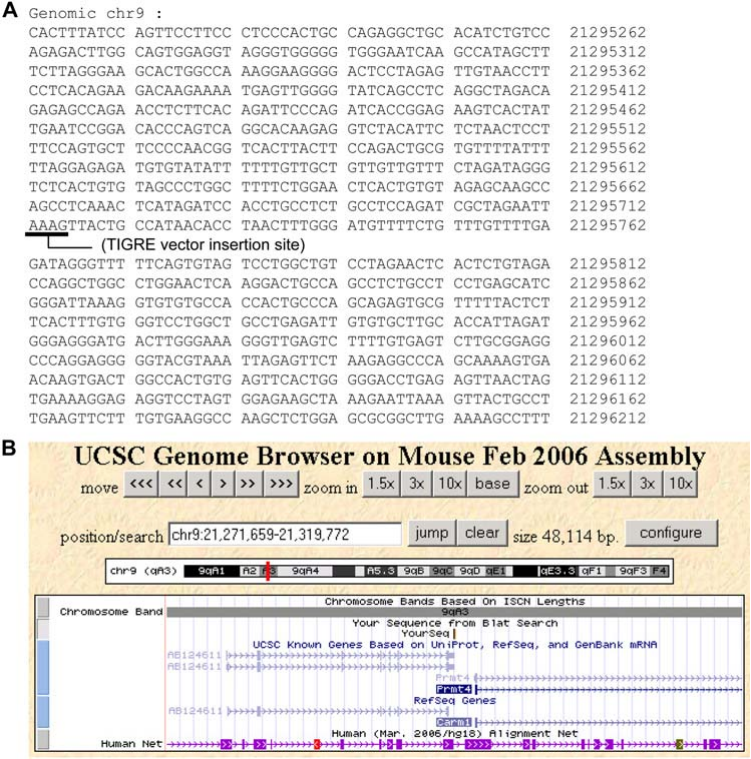
Chromosomal location of the T1 TIGRE locus. (A) Genomic sequences surrounding the T1 TIGRE locus. The underlined AAAG sequence was duplicated upon viral integration and the viral TIGRE vector was inserted exactly in between the duplication. (B) BLAT search of the UCSC Mouse Genome Browser (http://genome.ucsc.edu/cgi-bin/hgBlat) with genomic sequences (as in (A)) surrounding T1 revealed the localization of T1 locus to chr9 qA3. This panel is a screen shot of the BLAT search result. The location of the sequences used for the search is indicated by a vertical bar next to “YourSeq”. The insertion site is in between two genes: AB124611 and *Carm1* (alternative name *Prmt4*), and does not seem to disrupt either gene.

Using the splinkerette PCR method [16], we obtained genomic fragments covering either 5′ or 3′ junctions of the TIGRE vector insertion site in the T1 ES cell line. After sequencing the fragments, we determined the precise integration site of the viral TIGRE vector in the T1 TIGRE locus, which is located on chromosome 9. Genomic sequences surrounding the T1 TIGRE locus are shown in Figure 5A. Characteristic of retrovirus insertions, four nucleotides immediately adjacent to the insertion were duplicated and the viral TIGRE vector was inserted exactly in between the duplication. BLAT search of the UCSC Mouse Genome Browser with genomic sequences surrounding T1 revealed the localization of T1 locus to chr9 qA3 (Figure 5B). The insertion site is flanked by two genes: AB124611 and *Carm1*. The insertion site is located 3′ to the hypothetical gene AB124611 with unknown function and undetermined polyA site. The insertion site is also ∼1.5 kb upstream of the transcriptional start of *Carm1*. *Carm1* is ubiquitously expressed and *Carm1*−/− mice are embryonic lethal [17]. However, we have not observed overt developmental or other abnormalities in heterozygous or homozygous T1 TIGRE reporter lines TRE-lacZ or TRE-Luc (both with insulators), indicating that the viral insertion did not disrupt the nearby *Carm1* gene.

### KO Lines Produced from an ES Cell Library Mutagenized by a Retroviral Vector

**Figure 6:**
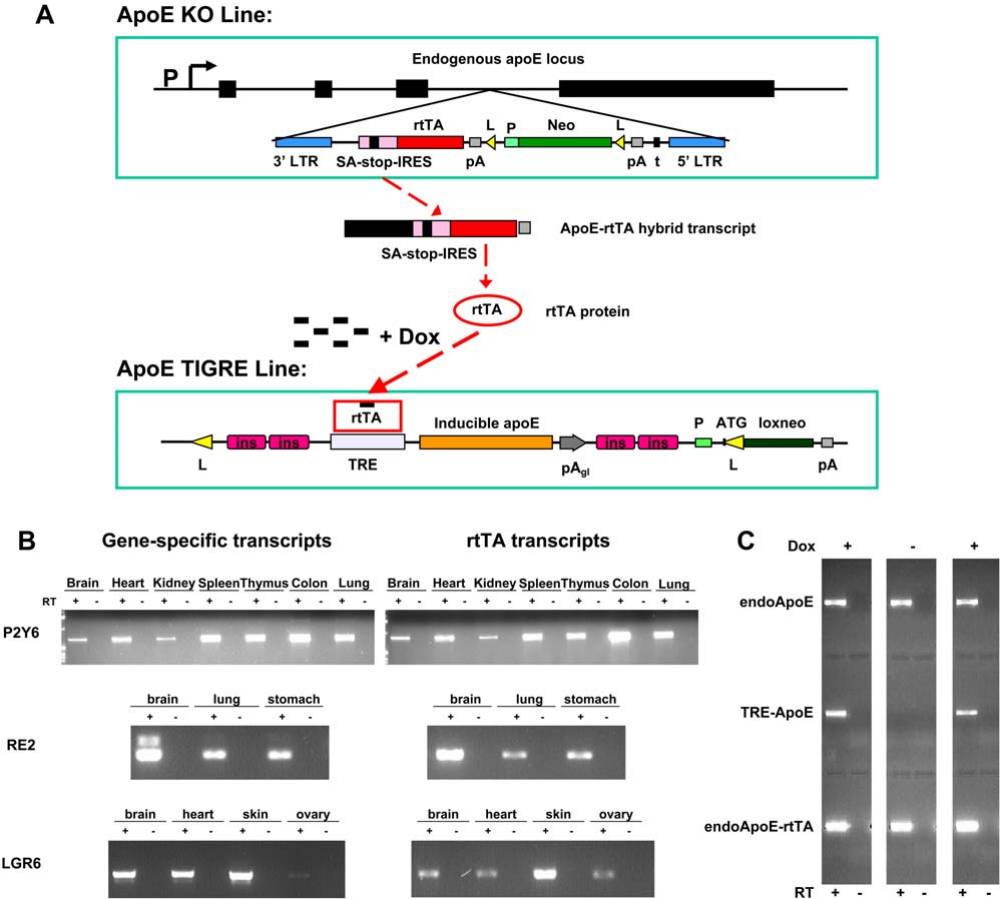
Characterization of gene regulation in iKO mice. (A) Construction and genomic structure of the ApoE iKO mice. Endogenous ApoE gene comprises of 4 exons. In the ApoE KO line, retroviral vector is inserted into the third intron of the ApoE gene, 205 bp upstream of the fourth exon (the largest coding exon). The retroviral vector contains the virus backbone (including 5′LTR and 3′LTR), a splice acceptor (SA) – stop codon (stop) – IRES cassette immediately followed by rtTA, a PGK promoter (P) driven neo selection marker flanked by two loxP sites (L), and a transcriptional terminator sequence (t). pA, polyadenylation sequence. From this locus, transcription initiated from the endogenous ApoE gene continues through rtTA to form an ApoE-SA-Stops-IRES-rtTA-polyA hybrid transcript in place of the full-length endogenous ApoE transcript. The rtTA protein is produced from this hybrid transcript through IRES-mediated translation, and in turn turns on the expression of the TRE-ApoE from the TIGRE locus only when Dox is present. The ApoE TIGRE locus contains an exogenous copy of ApoE cDNA driven by TRE and flanked by four copies of chicken β-globin insulator (ins) sequences, two on each side, and a PGK promoter (P) driven loxneo selection marker. (B) Side-by-side comparison of the expression of rtTA and each individual endogenous gene in various tissues by semi-quantitative RT-PCR. Three GPCR genes are shown here: P2Y6, RE2 and LGR6. Heterozygous mice are used so that the endogenous transcripts from the WT allele and the rtTA-containing hybrid transcripts from the KO allele can be amplified from the same RNA preps. Each RT-PCR reaction (RT+) has an RT- control run simultaneously to exclude any possibility of genomic DNA contamination. (C) Comparison of three transcripts by RT-PCR from the same RNA prep of the liver, the major site of normal ApoE production, of the ApoE+/−;TRE-ApoE mice – endogenous ApoE transcript (endoApoE), TRE-ApoE transcript from the TIGRE locus (TRE-ApoE) and ApoE-rtTA hybrid transcript (endoApoE-rtTA). Mice were fed either with or without Dox. As expected, expression of endoApoE and rtTA were independent of Dox. However, expression of TRE-ApoE was strictly dependent on Dox – it was undetectable in its absence and significantly expressed in its presence, indicating high degree of ApoE regulation achieved in the mice.

KO lines with target genes replaced by rtTA or tTA can be produced by any gene targeting or insertional mutagenic methods. To implement high-throughput production of iKO mice, we have utilized a large-scale insertional mutagenesis ES cell library developed in house for the KO production [18]. Figure 6A upper panel illustrates the structure of the retroviral vector used. In the particular case of ApoE, the virus is inserted in the third intron. The vector contains splice acceptor, stop codons, polyA signal and transcriptional terminator to ensure gene inactivation, which we confirmed to be the case by showing that ∼90% of isolated gene-specific ES clones were null alleles [18]. (The remaining were mostly knock-downs, and in nearly all these cases the retrovirus was inserted into 5′ UTR (exon or intron), suggesting that retroviral insertions upstream of the coding regions of genes should be avoided.) The vector also includes the rtTA gene immediately downstream of the splice acceptor, stop codons and internal ribosome entry site (IRES), so that rtTA protein can be synthesized from the ApoE-IRES-rtTA hybrid transcript.

To examine if random insertion of the retroviral vector containing rtTA into a gene results in rtTA expression reflecting the expression patterns of the inactivated gene, we compared rtTA transcription with the transcription of the endogenous gene by RT-PCR in different tissues for 26 different G protein coupled receptor (GPCR) KO lines generated from the library. Heterozygous mice carrying one allele of the intact endogenous gene and one allele interrupted by the rtTA-bearing retroviral vector were used to prepare total RNA samples from different tissues and amplify gene-specific transcript and rtTA transcript from the same RNA preps for side-by-side comparison. Figure 6B shows three examples of such comparison for genes P2Y6, RE2 and LGR6 respectively. The retroviral vector was inserted into a different part of each of the three genes – a 5′UTR intron of P2Y6, an intron within the coding region of RE2 and the 15^th^ coding exon of LGR6. In all three cases, rtTA expression profiles closely resemble those of the endogenous genes to be inactivated. Real-time qPCR for 16 tissues (Table S1) also showed high degree of correlation between rtTA and endogenous gene from tissue to tissue. Overall, out of the 26 lines examined, 19 lines showed good correlation between rtTA and the endogenous gene's expression (Table S2), i.e. rtTA is expressed in the tissues where the corresponding endogenous gene is expressed and the relative ratio across different tissues for each transcript also appears similar between rtTA and endogenous gene. In the remaining 7 lines in which rtTA was not expressed well, 5 lines had the retroviral vector inserted upstream of the first (and usually fairly large) intron of each gene. It has been recognized that the first intron (especially if it is large) could contain essential transcriptional regulatory elements, and thus insertions in this region might disrupt the transcriptional regulation and so should be avoided. Otherwise, our data showed that in the majority of insertional sites, rtTA could be expressed through the endogenous promoter upon integration via the retroviral vector.

### Tightly Regulated ApoE Expression and Blood Cholesterol Levels by Dox in the ApoE iKO Mice

To prove the concept of iKO system, we generated ApoE iKO mice by creating ApoE KO line and ApoE TIGRE line separately and breeding them together (Figure 6A), and attempted to model human conditions such as hypercholesterolemia and atherosclerosis. ApoE KO line was created by screening the mutant ES cell library as mentioned above. ApoE TIGRE line was created by inserting a TRE-ApoE transgene, flanked with 4 copies of chicken β-globin insulators, into the T1 TIGRE locus. RT-PCR analysis of heterozygous mice (ApoE+/−; TRE-ApoE) showed that expression of TRE-ApoE was strictly dependent on Dox (Figure 6C). Real-time qPCR using primers specific for the endogenous ApoE or TRE-ApoE (2 sets of primers for each gene) showed that endogenous ApoE mRNA level is very high (compared to 18s rRNA), and the TRE-ApoE mRNA level in the presence of Dox is ∼6.7 fold lower than the endogenous ApoE (ΔCt = 2.7 between TRE-ApoE and endogenous ApoE), while in the absence of Dox TRE-ApoE is >55,000 fold lower than the endogenous ApoE (ΔCt>15.8). This shows the TRE-ApoE induction by Dox is >8,000 fold.

**Figure 7:**
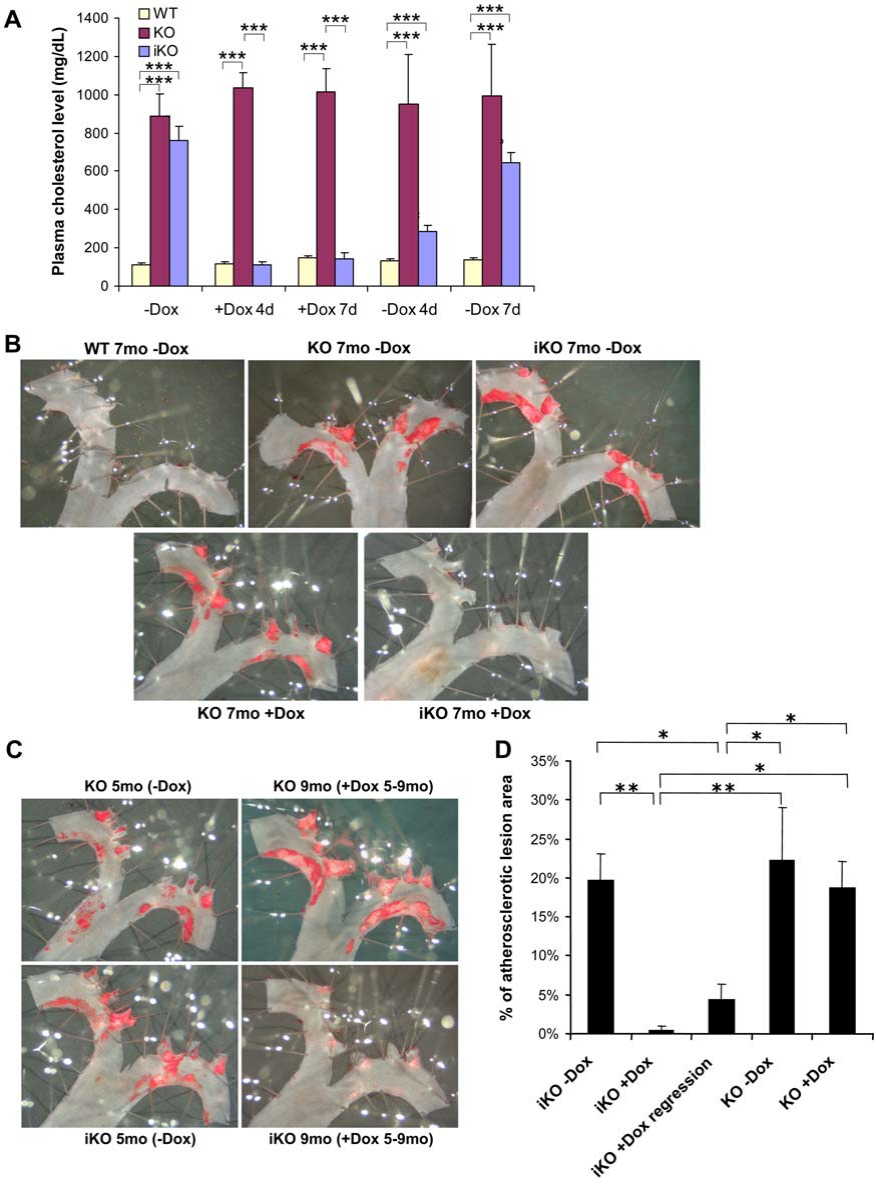
Plasma cholesterol levels and atherosclerotic lesion progression/regression regulated by Dox in ApoE iKO mice. (A) Plasma cholesterol levels in the ApoE iKO, KO and WT group mice in the absence and presence of Dox. (Littermate mice of various genotypes other than homozygous KO or iKO, i.e. ApoE+/+, ApoE+/−, ApoE+/+;TRE-ApoE and ApoE+/−;TRE-ApoE, all displayed normal and indistinguishable blood cholesterol levels, and never developed lesions under any treatment regime used in our study. Consequently they were lumped together as the “WT group”.) Plasma cholesterol levels were first measured in mice fed with normal food without Dox (−Dox) and both ApoE iKO and KO mice showed significantly higher cholesterol levels compared to WT group mice (p = 0.35 between iKO and KO, p<0.0001 between iKO and WT or between KO and WT, Student's t-test). The mice were then switched to Dox-containing food, and plasma cholesterol levels were measured again 4 days (+Dox 4d) and 7 days (+Dox 7d) later. Cholesterol level of ApoE iKO mice dropped to WT levels in less than 4 days while that of ApoE KO remained high (p<0.0001 between iKO and KO or between KO and WT, p = 0.86 between iKO and WT). Sometime later, some Dox-treated mice were switched back to normal food, and plasma cholesterol levels were measured again 4 days (−Dox 4d) and 7 days (−Dox 7d) after Dox withdrawal. Cholesterol level of ApoE iKO mice significantly elevated by day 4 (p<0.001 between iKO and WT, p<0.01 between iKO and KO) and approached pre-Dox treatment level by day 7 (p<0.0001 between iKO and WT, p = 0.13 between iKO and KO). ***P<0.001. (B) Atherosclerotic lesion progression. Aortas were stained with Sudan IV to visualize the lesions in red. The arch region of the aorta contains the most extensive areas of lesions and is shown here. A group of iKO, KO and WT mice were treated with Dox-containing food starting before the onset of lesions, and were compared with mice fed with normal food. At 7 months of age, aortas were dissected from these mice and lesions were examined. ApoE iKO mice showed extensive aortic lesions as the KO mice in the absence of Dox, and yet no lesions at all as the WT mice in the presence of Dox. (C) Atherosclerotic lesion regression. ApoE iKO and KO mice of 5 months of age were switched from normal food to Dox-containing food. Aortic lesions were examined before (at 5 months) and after (at 9 months) the Dox treatment. After 4 months of Dox treatment, the lesions in KO mice continued to grow, whereas in the iKO mice the lesions had regressed. (D) Quantification of the aortic atherosclerotic lesion areas in the arch region above the first intercostal artery, as expressed by the percentage of lesion areas versus the whole aortic area in this segment. All genotypes are matched with ages for different Dox treatment. Dox-treated groups are: iKO+Dox: Dox food started at 2–4 months of age (before the onset of atherosclerosis); iKO+Dox regression: Dox food started at 5–6 months of age (after the onset of atherosclerosis); KO+Dox: Dox food started at 2–4 months of age (before the onset of atherosclerosis). The results are compared using one-way ANOVA followed by Neuman-Keul's post hoc test. Both iKO+Dox and iKO+Dox regression groups had significantly reduced atherosclerotic areas compared to the remaining groups. *P<0.05, **P<0.01. The iKO mice without Dox, as well as KO mice either with or without Dox, all developed comparable areas of lesions (p>0.05 in all pair-wise comparisons). The iKO mice treated with Dox before the onset of atherosclerosis had nearly no lesions and were significantly different from the above groups (p<0.01 iKO+Dox versus iKO−Dox; p<0.01 iKO+Dox versus KO−Dox; p<0.05 iKO+Dox versus KO+Dox). The iKO mice treated with Dox after the onset of atherosclerosis had significantly reduced atherosclerotic areas compared to iKO mice without Dox or KO mice either with or without Dox (p<0.05 in all comparisons between iKO+Dox regression versus iKO−Dox, KO−Dox or KO+Dox).

Next, we carried out phenotypic analysis of homozygous ApoE iKO mice (ApoE−/−; TRE-ApoE), i.e. mice having both endogenous ApoE alleles inactivated and carrying TRE-ApoE in the TIGRE locus. We analyzed blood cholesterol levels in these mice in the absence and presence of Dox. Constitutive KO (i.e. ApoE−/− without TRE-ApoE in the TIGRE locus) and WT group mice that were littermates of iKO were used as controls. As shown in Figure 7A, in the absence of Dox the iKO mice had high cholesterol levels similar to that of the KO mice; in the presence of Dox, the iKO showed normal cholesterol levels, demonstrating that inducible expression of ApoE can lead to the reversion of the KO phenotype of hypercholesterolemia. When Dox was withdrawn, the cholesterol levels in the iKO mice rose again. These on/off switches occurred rapidly, within a few days after Dox administration or withdrawal.

### Dox-Regulated Atherosclerosis Progression and Regression in the ApoE iKO Mice

We examined the atherosclerotic lesion formation in the aortas of the ApoE iKO mice. As controls we used KO and WT group mice that were littermates of iKO. In the absence of ApoE protein, aortic atherosclerotic lesions start to form around 3–4 months of age and progress continually with time. One set of iKO, KO and WT group mice were treated with Dox-containing food throughout their life, and were compared with mice fed with normal food. Figure 7B shows the atherosclerotic lesions formed around the arch region of the aorta, as visualized by Sudan IV staining. By 7 months of age, extensive lesions had formed in KO mice, regardless of whether they were treated with Dox or not, whereas WT mice did not have any lesions in the absence (Figure 7B) or presence (data not shown) of Dox. The iKO mice developed extensive lesions in the absence of Dox (similar to KO mice), whereas in the presence of Dox no lesions had formed.

We further investigated what happens if ApoE protein expression is turned on after the atherosclerotic lesions have already formed. ApoE iKO and KO mice of 5 months of age were switched from normal food to Dox-containing food for the next 4 months. Aortic atherosclerotic lesions were examined before (at 5 months) and after (at 9 months) the Dox treatment. As shown in Figure 7C, at 5 months of age, both iKO and KO had developed similar levels of lesions. After 4 months of Dox treatment, the lesions in KO mice continued to grow, whereas in the iKO mice, the lesions had regressed nearly completely with only scar-like tissues remaining, suggesting that the lipid-containing foam cells have disappeared from the lesions. The results were verified by quantification and statistical analysis of the lesions by one-way ANOVA followed by Neuman-Keul's post hoc test (Figure 7D).

## Discussion

We have developed a reversible and inducible rescue system for gene KO in mice and have applied this method into the ApoE gene. Our system complements existing inducible gene expression approaches and provides certain advantages. The tamoxifen-dependent Cre-ERT2 [19],[20] recombination can drive inducible knockout of the endogenous gene, however, it is irreversible and the efficiency of tamoxifen inducibility throughout the body is yet to be demonstrated. The usefulness of the Tet-inducible system [21],[22] is critically dependent on the tightness of transcription induction and suppression. When either the Tet-transactivator or the target gene is randomly integrated into the genome, they are subjects to positional effects [23]. It is often necessary to screen through multiple transgenic lines every time a new transgene is introduced. Attempts to target both tTA and TRE together into an endogenous gene locus to achieve inducible activation and inactivation were successful in a few cases [24],[25],[26], but this method is not applicable to most other genes as the TRE is easily subject to activation from nearby enhancers independent from tTA. Our iKO technique utilizes each gene's own promoter to direct the expression of transcriptional activators, e.g. rtTA and tTA. Therefore it is not limited to certain tissues and available tissue-specific promoter driven transgenic lines. It could be applicable to any gene in any tissue. Dox-regulated expression can be turned on and off rapidly, i.e. within a few days, and at any time in development or adult. It also allows analysis of the effects of gene inactivation in the same animal. The TIGRE locus has been selected to confer little or no basal transgene expression throughout the body while maintaining high inducibility, enabling stringent control of the on/off switching of the target gene. Our system also has the flexibility to allow for further improvements. For example, in some cases it may be more desirable to put the genomic copy of the gene under TRE promoter into the TIGRE locus instead of the cDNA to more precisely mimic the expression of the endogenous gene. Also, the IRES we have used to drive rtTA translation may not work uniformly well in all tissues, and can be replaced by other approaches such as using the viral 2A-like sequences for bicistronic translation [27] or direct targeting of tTA/rtTA into the ATG start codon of the endogenous gene.

Unique chromosomal loci for predicted gene expression provide fundamental tools for genetic studies. The most widely used is the ROSA26 locus [28] in which ubiquitous gene expression is achieved by endogenous promoter activity of this locus. The TIGRE loci identified in this study allow a different mode of gene regulation – they were selected from hundreds of insertion sites for tight gene regulation by an exogenous promoter. Therefore, the TIGRE loci offer a platform for easy insertion of any gene in a tightly regulated locus, applicable to not only the tetracycline system but also other gene expression systems utilizing exogenous promoters such as constitutively active promoters, tissue specific promoters, or other inducible promoters regulated by reagents such as ecdysone [29], mifepristone [30] and streptogramin [31]. Recently a similar approach was also applied to a human fibrosarcoma cell line to pre-screen optimal integration sites for transgenes [32]. In addition, here we demonstrated that the stringency of the regulation at TIGRE loci is further enhanced by the incorporation of insulators, i.e. basal expression level was reduced to less than one luciferase molecule per cell without impairing inducibility (Figure 4C, D). Insulators were also shown previously to improve inducibility of randomly integrated TRE-reporters [33]. It should be noted that in our study the insulator effect was limited in many other loci (Figure 4A versus 4B), demonstrating the uniqueness of the TIGRE loci. The genomic location of the T1 TIGRE locus, which was most extensively used in our study including the ApoE iKO mice, was determined (Figure 5). Further manipulation of this locus would be possible to expand its application.

The stringent gene regulation is demonstrated in the ApoE iKO mice. It is known that regulation of blood cholesterol levels is very sensitive to the plasma ApoE protein levels. Even with the production of 3% of wild-type level of ApoE protein, the hypercholesterolemia and atherosclerosis phenotypes of the ApoE KO mice can be reversed [9]. Given this high sensitivity, the fact that our ApoE iKO mice in the uninduced state (i.e. in the absence of Dox) exhibit similarly high levels of cholesterol compared to ApoE KO mice indicates that there is hardly any expression of functional APOE. Reversing the KO at will is a particularly powerful approach in atherosclerotic regression studies. Dox-treatment of ApoE iKO mice results in expression of ApoE, marked reduction of plasma cholesterol levels, and regression of aortic atherosclerotic lesions. These findings are consistent with previous studies showing that aggressive lipid lowering or expression of ApoE can induce regression of pre-existing atherosclerotic lesion [7],[8],[34],[35]. It is becoming increasingly clear that lesion regression is regulated by a complex interplay between lipids, inflammation and the immune system [35]. The ApoE iKO mice will allow detailed studies on the roles of specific genes in these complex interactions.

The binary nature of the iKO system is inherently simple, with the KO line serving dual roles: it could be used as a constitutive KO or combined with the TIGRE line to produce an inducible and reversible iKO. The 10-million clone ES cell library we utilized [18] has been estimated to contain insertional mutations for >90% of genes, and individual ES clones with retroviral insertions in a specific target gene can be rapidly identified through a PCR pooling strategy and subsequently isolated in a streamlined process. The identification and modification of the TIGRE locus allows rapid insertion of any gene of interest via co-transfection with Cre. Therefore, each component of the binary system, the KO or the TIGRE line, is amenable for high-throughput production to generate inducible and reversible KOs for a large number of genes.

It should be noted that the iKO system may not be applicable in certain situations where highly stringent gene regulation is required. For example, even though the system had enough stringency in low basal activity and high induction, the induced gene expression level is usually not exactly the same as the endogenous gene's level and this could be a problem for genes that are highly sensitive to gene dosage effect or show haploid deficiency. The kinetics of the system (days) may be also too slow for some developmental problems where transient expression of developmental genes are critical, although it should be noted that it could still work well in carefully thought-out developmental studies (as in [24]). In addition, it has been reported that rtTA often can not induce sufficient gene expression in the brain, at least partially due to developmental inactivation of the TRE promoter in neurons [36]. It appears that the tTA (Tet-off) system is better suited for the use in brain [14], as substantial β-gal induction was observed in brain with tTA (Figure 4F).

Our results of the ApoE iKO mice and the quantitative data using lacZ and luciferase reporters suggest that the iKO system could be a useful tool in addressing a variety of biological questions. The two components of the iKO system can be independently modified, and pairing of their different forms can generate numerous combinations. In KO lines, genes with unique expression patterns are tagged with a transcription transactivator, which can control, in an inducible fashion, the expression of a variety of genes derived from TIGRE lines, enabling a number of additional applications in specific types of tissues or cells. Those include: 1) introducing mutant forms of the target gene into the TIGRE locus for better functional probing of different domains, splice variants, post-transcriptional modifications (e.g. phosphorylation), or modeling human diseases; 2) humanizing target genes by placing a human ortholog of the mouse gene under TRE control, which can facilitate drug efficacy studies; 3) placing a cytotoxic gene under TRE control to allow inducible cell-type specific ablation; 4) introducing a marker gene such as GFP, protein interacting probe, or transneuronal tracer, into the TIGRE locus for cell-type specific tagging, functional analysis, isolation of specific cell population, or mapping neuronal networks; 5) combining with recently developed RNAi techniques [37],[38],[39] to down-regulate any genes of interest in a tissue-specific and inducible manner; 6) re-engineering the TIGRE locus to place a TRE-driven Cre, and combining it with a tissue-specific rtTA or tTA line and floxed target genes to achieve inducible region-specific gene knockout [40].

## Materials and Methods

See Text S1 online for a more detailed description of the methods.

### Screening and Isolation of ES Clones with Insertions in Target Genes

Construction of ES cell library infected with the retrovirus and screening and isolation of ES clones with genes of interest inactivated by the viral insertion is described previously [18]. Specifically for ApoE, insertions were found by nested PCR analysis of the ES DNA using two vector-specific and gene-specific primer pairs. The ApoE-specific primers are antisense and located in the fourth exon. Several independent retroviral insertions in the ApoE gene were identified. PCR fragments were sequenced to confirm insertion into the gene. A library tube with a clone of interest identified by the PCR also contains a few hundred other ES cell clones. The sole desired clone was isolated from the mixture by three rounds of cell sorting and growing followed by PCR using the same pair of primers to identify positive clones. Additional PCRs using ApoE primers located in the third intron flanking the viral insertion site were conducted to confirm the precision of the insertion and integrity of the genomic sequence of ApoE.

### Generation of TIGRE ES Clones Containing Target Genes

Full length cDNAs for the coding sequences of target genes were cloned into the TIGRE-targeting vector containing TRE, insulators, a PGK promoter and a pair of loxP sites (Figure 5A lower panel). The TIGRE-targeting vectors were co-transfected with a Cre-expressing plasmid into the neo-sensitive ES cells carrying the minimal TIGRE locus with a single loxP site and the promoterless, ATGless loxneo marker. When the TIGRE-targeting vector is integrated into the TIGRE locus through Cre/lox-mediated recombination, neo-resistance is restored to the ES cells by the addition of PGK promoter and in-frame fusion of ATG to the loxneo marker. Correctly integrated ES clones were identified and confirmed by PCR screening and southern blot analysis.

### Animal Production and Maintenance

ES cell clones were injected into blastocysts of C57BL/6J mice following standard techniques. Chimeric mice were bred with C57BL/6J mice to test germline transmission and generate heterozygous mice. Mice from the corresponding KO lines and TIGRE lines were crossed with each other to produce inducible KO mice according to the scheme shown in Figure 1B. All mice used for studies in this paper were in a mixed genetic background of 50% 129S1/SvImJ and 50% C57BL/6. For the ApoE KO line, southern blot using rtTA coding sequence as probe confirmed the correct insertion of the retroviral vector into the endogenous ApoE gene. It also revealed an additional retroviral insertion somewhere else in the genome. The additional insertion was selectively bred out, and the ApoE iKO colony was maintained with a single retroviral insertion at the ApoE locus. MMTV-tTA, P_CAMKII_-tTA and P_NSE_-tTA mice were purchased from The Jackson Laboratory (Bar Harbor, ME). All experimental procedures were approved by the Institutional Animal Care and Use Committee of NCI and Nura, Inc. in accordance with NIH guidelines.

Dox was administered to the mice through Dox-containing food, which was custom made by Bio-Serv (Frenchtown, NJ) to contain 2 g Dox per kilogram of food. The nutrition content of the Dox food (e.g. 19% protein and 8.6% fat) was very similar to the regular diet (20% protein, 9% fat) used in our colony. Therefore the switching of food types did not result in change of cholesterol levels in mice.

## Supporting Information

Figure S1 Cre-mediated introduction of the insulator sequence into the LacZ gene at the TIGRE loci.
(0.91 MB TIF)

Figure S2 Cre-mediated introduction of the luciferase gene into the TIGRE loci.
(1.43 MB TIF)

Figure S3 Effect of Transrepressor on basal activity.
(0.11 MB TIF)

Table S1 Comparison of rtTA and endogenous gene by real-time qPCR in 3 KO lines.
(0.08 MB DOC)

Table S2 Comparison of rtTA and endogenous gene by RT-PCR in 26 KO lines.
(0.07 MB DOC)

Text S1 Supporting Methods and Supporting Figure Legends.
(0.12 MB DOC)
