# Supplementary figures and images for: An Inducible and Reversible Mouse Genetic Rescue System
Source: PLoS Genet. 2008 May 9;4(5):e1000069. doi: 10.1371/journal.pgen.1000069 (PMC2346557; doi:10.1371/journal.pgen.1000069)

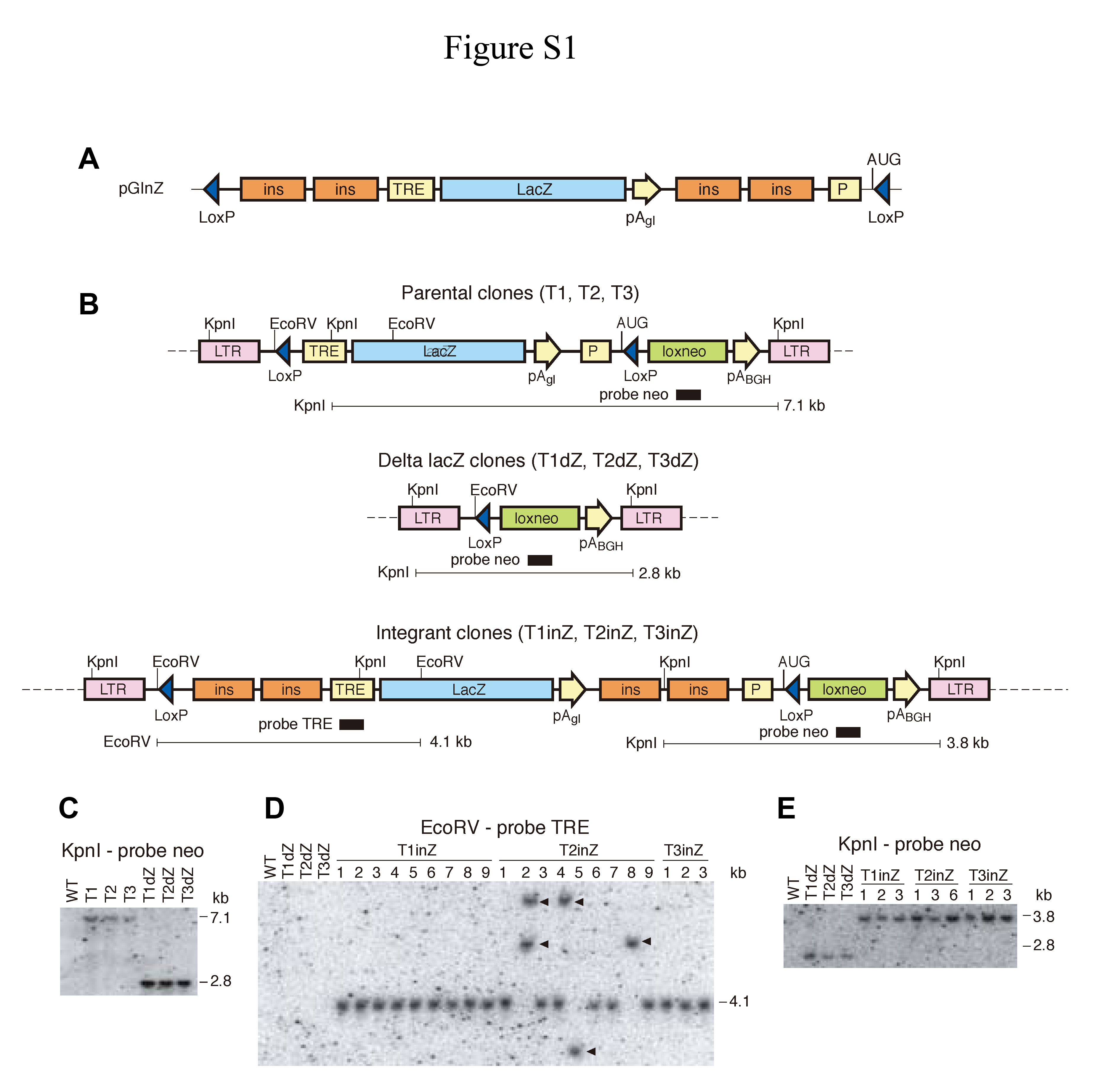

Supplement: Figure S1 — Cre-mediated introduction of the insulator sequence into the LacZ gene at the TIGRE loci. (0.91 MB TIF) [file pgen.1000069.s001.tif]

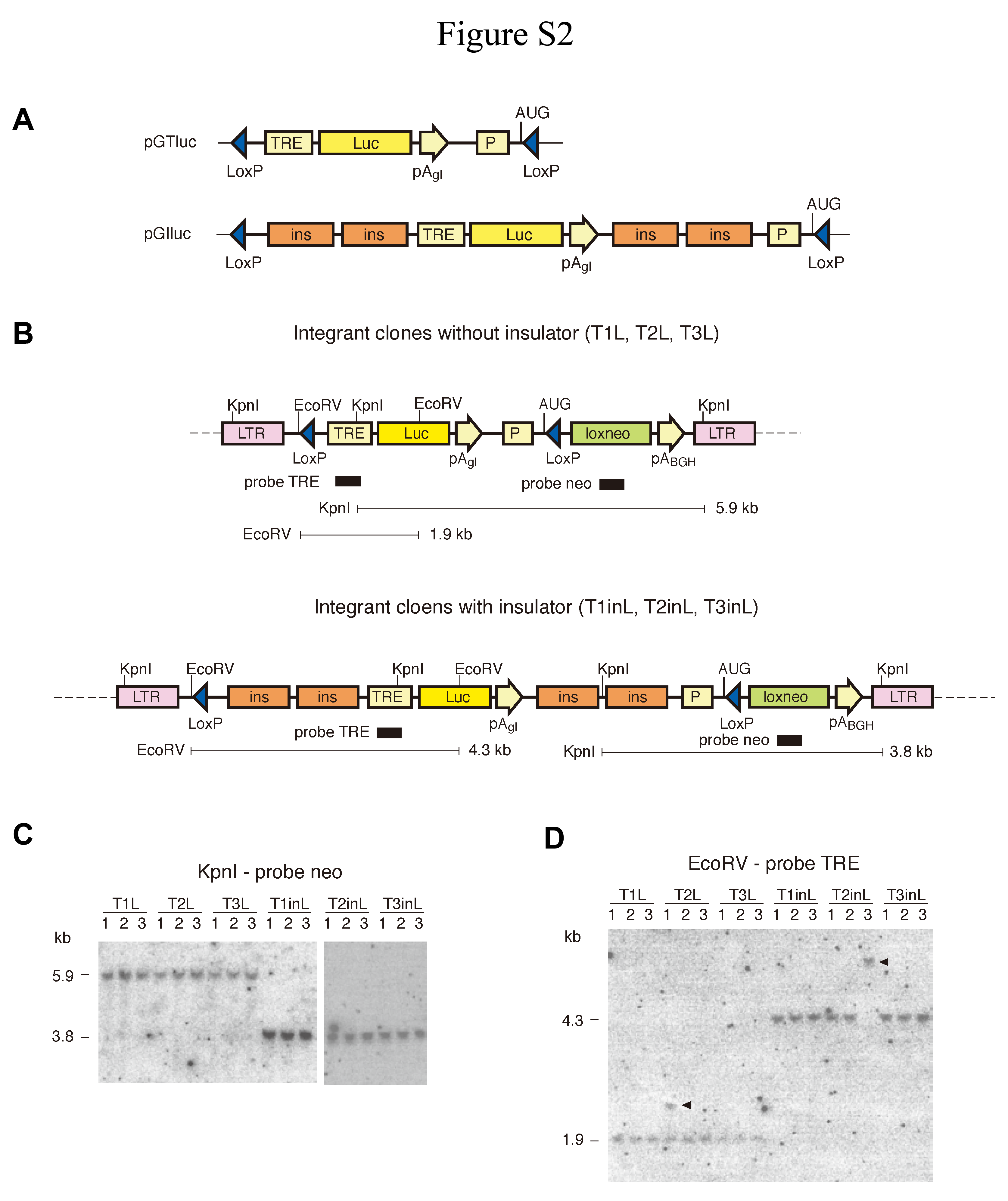

Supplement: Figure S2 — Cre-mediated introduction of the luciferase gene into the TIGRE loci. (1.43 MB TIF) [file pgen.1000069.s002.tif]

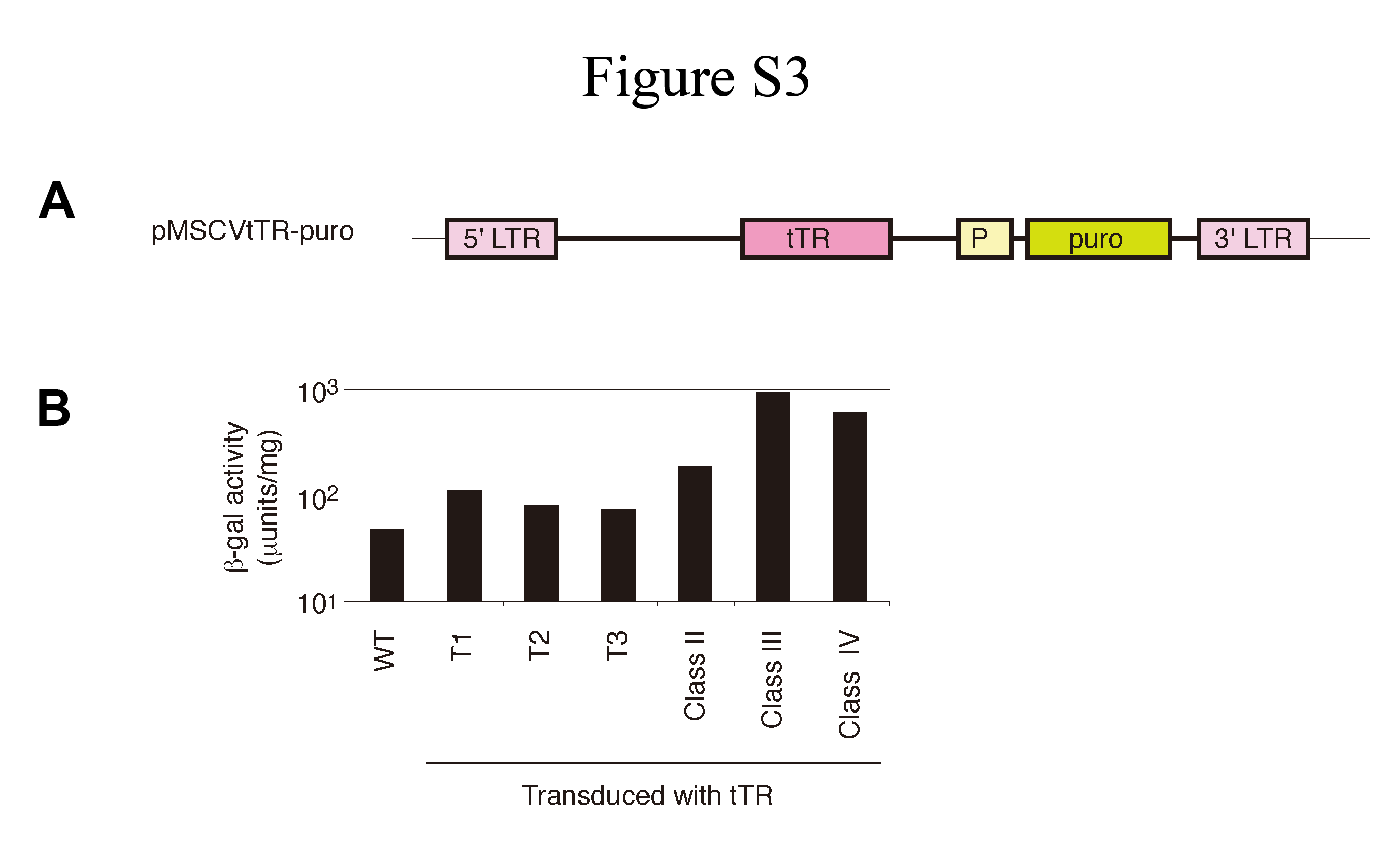

Supplement: Figure S3 — Effect of Transrepressor on basal activity. (0.11 MB TIF) [file pgen.1000069.s003.tif]
