# Supplementary material for: An Inducible and Reversible Mouse Genetic Rescue System
Source: PLoS Genet. 2008 May 9;4(5):e1000069. doi: 10.1371/journal.pgen.1000069 (PMC2346557; doi:10.1371/journal.pgen.1000069)
Supplement: Table S1 — Comparison of rtTA and endogenous gene by real-time qPCR in 3 KO lines. (0.08 MB DOC) [file pgen.1000069.s004.doc]

**Table S1.** Side-by-side comparison of the expression of rtTA and each individual endogenous gene in various tissues of heterozygous mice (P2Y6, RE2, LGR6) by real time qPCR.

| Tissue | ΔCt rtTA | ΔCt P2Y6 | ΔΔ | ΔCt rtTA | ΔCt LGR6 | ΔΔ | ΔCt rtTA | ΔCt RE2 | ΔΔ |
| --- | --- | --- | --- | --- | --- | --- | --- | --- | --- |
| Brain | 20.9 | 18.3 | 2.6 | 23.3 | 19.9 | 3.4 | 19.1 | 16.5 | 2.6 |
| Prostate | 22.4 | 22.1 | 0.3 | no exp | no exp |  | 25.2 | 23.6 | 1.6 |
| Thyroid | 24.3 | 22.6 | 1.7 | 25.5 | 22.9 | 2.6 | 27.1 | 24.7 | 2.4 |
| Lung | 19.5 | 15.7 | 3.8 | 24.5 | 21.7 | 2.9 | 24.6 | 21.9 | 2.7 |
| Bone | 23.5 | 23.5 | 0.0 | 24.8 | 23.1 | 1.7 | 27.9 | 25.3 | 2.6 |
| Skin | 24.1 | 22.1 | 2.0 | 19.8 | 16.5 | 3.2 | 25.4 | 20.4 | 4.9 |
| Heart | 20.1 | 17.6 | 2.4 | 23.4 | 19.1 | 4.3 | n/a | n/a |  |
| Thymus | 18.4 | 15.3 | 3.1 | 25.0 | 23.4 | 1.6 | n/a | n/a |  |
| Spleen | 19.2 | 17.0 | 2.3 | 26.2 | 23.4 | 2.7 | no exp | 25.3 | >5 |
| Stomach | n/a | n/a |  | 29.0 | 25.9 | 3.1 | 24.7 | 19.9 | 4.8 |
| Testis | 23.4 | 22.3 | 1.2 | 21.7 | 22.5 | -0.8 | n/a | n/a |  |
| Colon | 17.3 | 15.2 | 2.2 | no exp | no exp |  | 27.1 | 22.8 | 4.3 |
| Kidney | 21.7 | 19.2 | 2.5 | n/a | n/a |  | no exp | no exp |  |
| Spinal cord | n/a | n/a |  | 25.2 | 22.7 | 2.4 | 23.3 | 20.4 | 2.9 |
| Intestine | 24.9 | 23.4 | 1.5 | no exp | no exp |  | no exp | 26.7 | >4 |
| Liver | n/a | n/a |  | no exp | no exp |  | n/a | n/a |  |

## The ΔCt is the difference in number of cycles of signal appearance between each gene and 18S. Lower ΔCt means higher expression level. "No exp" (no expression) means that there is no gene-specific signal in 40 cycles. Discrepancies in expression are marked in red.
