# Supplementary material for: An Inducible and Reversible Mouse Genetic Rescue System
Source: PLoS Genet. 2008 May 9;4(5):e1000069. doi: 10.1371/journal.pgen.1000069 (PMC2346557; doi:10.1371/journal.pgen.1000069)
Supplement: Table S2 — Comparison of rtTA and endogenous gene by RT-PCR in 26 KO lines. (0.07 MB DOC) [file pgen.1000069.s005.doc]

**Table S2. Side-by-side comparison of the expression of rtTA and each individual endogenous gene in various tissues of heterozygous mice by RT-PCR. Out of the 26 KO lines examined, 19 show good correlation between endogenous and rtTA transcripts and 7 do not. Retroviral insertion site was identified for each KO line.**

| Gene | Insertion site | Endogenous transcript level | rtTA transcript level | Tissues examined |
| --- | --- | --- | --- | --- |
| ***Lines that correlate:*** | |  |  |  |
| GPR85 | Intron 1 or 2 (5' UTR)(1.2kb) | medium high | medium high | brain |
| P2Y6 | Intron 1 or 2 (5' UTR)(0.6kb) | low to high | low to high | multiple |
| GPR19 | Intron 1 (5' UTR)(17kb)(very close to splice donor) | medium high | medium | brain |
| CYSLT2 | Intron 5 (5' UTR)(1.9kb) | low | low | stomach, lung |
| PG63 | promoter? (250bp upstream of putative transcription start) | medium high | medium high | brain |
| RE2 | Intron 1 (within coding)(3.7kb) | multiple | multiple | multiple |
| Adora2b | Intron 1 (within coding)(16kb) | medium or low | medium or low | brain, heart |
| GALR2 | Intron 1 (within coding)(1.1kb)(very close to splice donor) | none | none | brain |
| GPR54 | Intron 2 (within coding)(1.1kb) | medium | medium | brain, lung |
| NTR2 | Intron 3 (within coding)(1.3kb) | high | high | brain |
| ApoE | Intron 3 (within coding)(0.4kb) | high | high | liver, brain |
| GPR63 | The only exon known (coding)(5' and 3' UTR unknown) | medium high | high | brain |
| P2Y5 | The only exon known (coding)(5' and 3' UTR unknown) | medium high | medium | brain, kidney, lung, skin |
| GPR20 | The only exon known (coding) | medium or low | medium low | stomach, intestine |
| SSTR5 | The only exon known (coding) | medium low | medium low | brain |
| GPR44 | The only exon known (coding)(5' and 3' UTR unknown) | low or none | low or none | stomach, lung |
| V1aR | Exon 1 (coding) | low | low | brain |
| HRH3 | Exon 2 (2nd coding exon) | high | high | brain |
| LGR6 | Exon 15 (largest exon, contains 7TM) | multiple | multiple | multiple |
| ***Lines that don't correlate:*** | |  |  |  |
| ADMR | Intron 1 (5' UTR)(0.5kb) | medium to high | low or none | brain, heart, liver, lung |
| GPR56 | Intron 1 (5' UTR)(>25kb)(very close to splice donor) | medium high | low or none | brain |
| TRHR | Intron 1 (5' UTR)(0.6kb) | medium low | low or none | brain, pituitary |
| F8 | Intron 1 (within coding)(11kb)(very close to splice donor) | medium high | low or none | liver, spleen |
| GLP2R | Intron 1 (within coding)(5.8kb)(very close to splice donor) | medium | none | brain, colon, stomach |
| MC1R | The only exon known (3' UTR) | medium high | none | skin |
| GPR39 | Exon 1 (coding)(5' and 3' UTR unknown) | medium high | low | stomach, intestine |
