# Supplementary material for: An Inducible and Reversible Mouse Genetic Rescue System
Source: PLoS Genet. 2008 May 9;4(5):e1000069. doi: 10.1371/journal.pgen.1000069 (PMC2346557; doi:10.1371/journal.pgen.1000069)
Supplement: Text S1 — Supporting Methods and Supporting Figure Legends. (0.12 MB DOC) [file pgen.1000069.s006.doc]

## Text S1

**Supporting Methods**

**Plasmid construction.** pRTonZ contains expression unit of the lacZ gene and the neo gene. The lacZ gene has a nuclear localization signal of SV40 T antigen (Pro-Lys-Lys-Lys-Arg-Lsy-Val) [1] which allows to distinguish lacZ-gene derived ß-galactosidase (ß-gal) activity from endogenous activity. It was inserted between the tetracycline-responsive element (TRE) and a rabbit ß-globin polyA addition signal of pUHG10-3 (gift from Dr. H. Bujard at Universitat Heidelberg), and a loxP site was placed upstream of TRE. The neo expression unit, designated loxneo, was generated by introducing an in-frame loxP sequence downstream of the initiation codon of the neo gene of pPGKneobpA [2]. Sequence at the 5’ end of the loxneo gene is ATG GGA ATA ACT TCG TAT AGC ATA CAT TAT ACG AAG TTA TAT GCT AGC. The first three nucleotides are initiation codon and the underlined nucleotides are loxP site. The vector backbone is the Moloney murine leukemia virus (MoMLV)-based retroviral vector pGen^-^ whose 3’ LTR lacks the viral enhancer to avoid misregulation of flanking genes [3].

To generate pGInZ, pGTluc, and pGIluc, an intermmediate plasmid pGTnZ was generated by subcloning a 5.4-kb NheI-SalI fragment of pRTonZ containing loxP-TRE-lacZ-polyA addition signal-PGK promoter-ATG-loxP into the NheI-SalI sites of pUC19. pGTluc was generated by replacing the lacZ gene of pGTnZ by a luciferase gene. pGInZ and pGIluc were created by introducing two copies of 1.2-kb chicken ß-globin insulator sequences of pJC13-1 [4] at each end of lacZ expression unit of pGTnZ, and luciferase expression unit of pGTluc, respectively.

pMSCVtTR-puro was generated by cloning the 1.1-kb EcoRI fragment of pCMV-tetR(B/E)-KRAB [5] containing tetracycline-controlled transrepressor region into the EcoRI site of pMSCVpuro (Clontech Laboratories, Inc., Mountain View, CA).

**Virus preparation and infection of ES cells.** Retrovirus stocks were prepared as described previously [6] by transient cotransfections of 293T cells with the retroviral vector pRTonZ, the MoMLVgag-pol expression vector pHIT60 [7] and the vesicular stomatitis virus G protein expression vector pHCMV-G [8]. CJ7 ES cells [9] were maintained as described previously [10] and infected at less than 0.1 of multiplicity of infection to avoid multiple integration of the retrovirus vector. ES cells were selected in G418 for 7 days. Resistant colonies were transferred onto mitomycin C-treated STO feeder cells [10] and expanded for further analysis.

**Transfection of TIGRE ES cells for Cre-mediated recombination.** For Cre-mediated excision, 2 x 10^7^ ES cells were electroporated by Gene Pulser (BioRad) with 20 µg of Cre expression vector pIC1-Cre [11] at 250 V and 500 µF using a 0.4 cm cuvette. The ES cells were plated on three 10cm dishes containing mitomycin C-treated STO cells, and replated sparsely (200, 500 or 1000 cells/10 cm dish) 48 hours after electroporation. Colonies were picked up 7 days after electroporation, and plated in duplicate wells, one with and the other without G418. G418-sensitive clones were identified and expanded to verify the recombination pattern by Southern blot analysis. Clones showing successful excision were used for the integration step. For the Cre-mediated integration step, ES cells were electroporated under the same condition as described above with 20 µg of Cre expression vector pBS185 [12] and 20 µg of the targeting vector (pGInZ, pGTluc or pGIluc). ES cells were plated on five 10 cm dishes with mitomycin-treated STO cells, and treated with G418 starting at 24 hours post-transfection. G418-resistant clones were transferred on mitomycin C-treated STO cells 8 days post transfection and expanded for further analysis. For Southern blot analysis, 10 µg of genomic DNA were digested by restriction enzymes, fractionated on 0.6% agarose gels and transfered to nylon membrane. Hybridization and washing were done using standard procedures.

**Real-time PCR quantitation of tTA mRNA in mouse tissues.** Tissue samples were homogenized in RNAzolB (TEL-TEST, Friendswood, TX), and total RNA was purified using manufacturer's protocol. First-strand cDNA was synthesized using the TaqMan reverse transcription reagents (PE Biosystems, Foster City, CA). In brief, 100 ng of total RNA from each tissue was reverse-transcribed by incubation in 50 mM KCl, 10 mM Tris-HCl (pH 8.3), 5.5 mM MgCl_2_, 500 µM of each dNTP, 2.5 µM random hexamer, 0.4 units/µl RNase inhibitor, 1.25 units/µl MultiScribe reverse transcriptase in a total volume of 10 µl. The mix was incubated at 25 °C for 10 min, 48 °C for 30 min, followed by enzyme denaturation at 95 °C for 5 min. Reactions containing no reverse transcriptase were used as negative control. mRNAs for tTA and glyceraldehyde phosphate dehydrogenase (GAPDH) were quantified by real-time PCR on ABI PRISM 7700 Sequence Detection System (PE Biosystems) using molecular beacons [13]. The sequence for tTA-specific beacon was 5’ CGGCCACGAGTTTGAGCAGATGTTTACCTGGCCG 3’ and the sequence for GAPDH-specific beacon was 5’ CGGCCAACATGTTCCAGTATGACTCCACTGGCCG 3’. Complimentary sequences are underlined. The molecular beacons have the reporter dye, FAM (6-carboxy-fluorescein) at the 5’ end, and the quencher dye, (DABCYL 4-(4’-dimethylaminophenylazo)benzoic acid) at the 3’ end. For the amplification of tTA transcript, the following primers were used: Forward primer was 5’ CCCTACGGCGCTCTGGATATGGC 3’ located in tTA coding region, and reverse primer was 5’ GAGACAGCACAACAACCAGCACGTTG 3’, located in the polyA addition signal of tTA expression unit. For the amplification of GAPDH transcript, the following primers were used. Forward primer was 5’ Ggcaaagtggagattgttgccatcaac 3’, and reverse primer was 5’ tctcggccttgactgtgccgttg 3’. Real-time PCR of tTA and GAPDH transctipts were performed in separate tubes using the TaqMan PCR core reagent kit (PE Biosystems). The reaction mix contained 0.25 µM of molecular beacon, 0.4 µM of each primer, 0.2 mM dATP, 0.2 mM dGTP, 0.2 mM dCTP, 0.2 mM dUTP, 3.5 mM MgCl_2_, 2.5 units of AmpliTaq Gold DNA polymerase in 1x TaqMan Buffer A. 10 ng of cDNA was used from each tissue, and 100 ng, 10 ng, 1 ng and 0.1 ng of cDNA aliquots from spleen were used as standards. Each PCR was performed in duplicate. Amplification conditions were 94 °C for 10 min for 1 cycle, followed by 45 cycles of 94 °C denaturation for 15 sec, 55 °C annealing for 1 min and 72 °C polymerization for 30 sec. Linearity of standard curve with correlation coefficient better than 0.99 was obtained. Quantity of tTA and GAPDH transcript in each tissue was obtained from the standard curve. Amount of tTA transcript was divided by the amount of GAPDH transcript for normalization. Values were multiplied by 1,000 for better representation.

**ß-gal staining.** To analyze bacterial ß-gal activity of ES cells in situ, cells were fixed with 0.05 % glutardehyde in PBS for 10 min, washed by PBS three times, and stained for 24 hours at 37 °C in a solution containing 1 mg/ml 4-chloro-5-bromo-3-indolyl-ß-D-galactopyranoside (X-gal, Sigma, St. Louis, MO), 1 mM MgCl_2_, 10 mM K_3_Fe(CN)_6_, and 10 mM K_4_Fe(CN)_6_·3H_2_O.

**ß-gal and luciferase assays in ES cells**. The same transfection conditions were used to determine the induction levels of ß-gal and luciferase activities in ES cell clones carrying the lacZ gene or the luciferase gene under the control of TRE. ES cells were passaged on gelatin-coated plates without STO cells three times prior to transfection, so that the endogenous ß-gal activity of STO cells will not affect the assay. 1x10^5^ ES cells were plated on gelatin-coated 24 well plates the day before transfection. Next day, 0.2 µg of tTA expression vector pUHD15-1neo [14] was transfected using 1.6 µl of Lipofectamine (LifeTechnologies, Gaithersburg, MD) per well together with 0.2 µg of luciferase expression vector or 0.2 µg of bacterial ß-gal expression vector as an internal control for transfection efficiency. The medium was replaced 20 hours post-transfection. Cells were trypsinized 48 hours post-transfection, washed once by PBS, and suspended in 23 µl of following lysis buffer: 100 mM potassium phosphate, pH 7.8, 0.2% TritonX-100, 0.5 mM DTT. Cell lysates were centrifuged at 12,500 x g for 10 min and 2 – 10 µl of supernatants were assayed in a MLX multi-well format luminometer (Dynex Technologies, Chantilly, VA). In case transfection was not needed, cell extracts were prepared by the same procedure from a confluent well of 24 well plates. Chemiluminescent reporter assay system Galacto-Star (Tropix, Bedford, MA) was used to quantitate ß-gal activity according to manufacture’s instruction. Luciferase activity was measured using the Luciferase Assay Kit (Tropix) according to manufacturer’s instructions. Protein concentrations were determined using the Bradford assay (Bio-Rad Laboratories, Hercules, CA).

**Estimation of the number of luciferase molecules per ES cell.** Luciferase activities of ES cell extracts were measured as described above and compared with standard preparations of recombinant luciferase (Promega, Madison, WI). The number of luciferase molecules in each extract was further calculated based on the molecular weight of luciferase. This number was divided by the number of ES cells contributing to each extract, leading to the number of luciferase molecules per cell. Two independent ES clones were analyzed in each integration site and mean values were calculated.

**ß-gal assay of mouse tissues.** ß-gal activity of mouse tissues were measured as described previously [15]. Mice were killed by carbon dioxide overdose. Tissue samples were homogenized in 1 ml of the following buffer: 100mM potassium phosphate, pH 7.8, 0.2% TritonX-100, 0.5 mM DTT, 0.2 mM phenylmethylsulfonyl fluoride (Roche Molecular Biochemicals, Indianapolis, IN), and 5 µg/ml leupeptin (Roche Molecular Biochemicals). The last three components were added just before use. The homogenates were centrifuged at 12,500 x g for 10 min, and the supernatants were heated at 48 °C for 60 min to inactivate the endogenous eukaryotic ß-gal [15,16]. The samples were centrifuged again at 12,500 x g for 20 min, and 2-10 µl of supernatant fluid was assayed for ß-gal activity using Galacto-Star (Tropix) as described above.

**RT-PCR.** Tissues were dissected and immediately stored in RNALater (Ambion, Austin, TX) at 4^o^C until RNA isolation. RNA was isolated from homogenized tissues by phenol extraction and LiCl precipitation using Totally RNA kit (Ambion). RNA was treated with DNase I (Ambion) for 1 h at 37^o^C. The quantity and quality of total RNA preps were assessed by UV spectrometry and gel electrophoresis (visual examination of 18S and 28S rRNA bands). Semi-quantitative RT-PCR was conducted in the following way. Equal amounts (~100 ng) of total RNA were used in each reverse transcription (RT) reaction using a Super-Script First Strand Synthesis kit (Invitrogen, Carlsbad, CA) with random primers. Each reaction was run in duplicate with reverse transcriptase (RT+) or without (RT-) to control for any potential genomic DNA contamination. cDNA (from 1/20^th^ of the RT reactions) was amplified for 30 cycles (94^o^C for 30s, 56^o^C for 40s, 72^o^C for 90s) with gene specific primers. Sometimes PCR reactions with less than 30 cycles were also run to make sure that PCR amplification was not saturated. As an internal control, PCR of GAPDH gene was run on the same RT reactions.

**Real-time qPCR.** Real-time qPCR was conducted using cDNA from the above RT reactions with gene specific primer pairs as well as a positive primer pair of 18S. The reactions are carried out in final volume of 25 μl that contains 12.5 μl of SYBR Green PCR Master Mix (Applied Biosystems, Foster City, CA), 0.5 μM of each primer, and ~20-50 ng cDNA. Each sample was run in triplicates. The following standard cycle was employed: 95^o^C for 10 min, followed by 40 cycles of 94^o^C 15 sec - 56^o^C 15 sec - 72^o^C 1 min in 7900HT Fast Real-time PCR system (Applied Biosystems). On completion of a real-time qPCR experiment, thermal denaturation profile of the resulting amplicon was determined in order to make sure that the same specific amplicon is detected in different samples. Difference in number of cycles needed to reach a threshold level of fluorescence with gene-specific primers as compared with 18S primers (ΔCt) was used as measure of relative mRNA abundance.

**β-galactosidase staining of tissue sections.** Mice were perfused with saline solution for 2-5 min, and then with 4% paraformaldehyde solution for 15min. Tissues were dissected out and further fixed in 4% paraformaldehyde for 1hr at room temperature. Fixed tissues were rinsed in PBS and cut into 50μm or 100μm sections using a vibratome. Tissue sections were washed in rinse buffer (100mM sodium phosphate, pH7.3, 2mM MgCl2, 0.01% sodium deoxycholate, 0.02% NP-40) for 3 times, 10 min each time. They were then soaked in freshly made staining solution containing X-gal (staining solution is rinse buffer plus 5mM potassium ferricyanide, 5mM potassium ferrocyanide and 1mg/ml X-gal) and incubated at 37^o^C for 16 hours in the dark. After staining, tissue sections were placed in 70% ethanol for storage.

**Cholesterol assay.** After fasting for 5-10 hours, mice were bled from tail tips or retro-orbital cavities using heparinized pipettes to obtain 50-100μl blood per mouse. Blood was centrifuged at 2,500rpm for 5min at 4^o^C, and plasma (top layer) was transferred to a new tube. Plasma cholesterol levels were measured using the Infinity Cholesterol reagent (Thermo Electron Corporation, Waltham, MA). Specifically, 4.5μl plasma sample was mixed with 0.9ml Infinity Cholesterol reagent and incubated at 37^o^C for 5min. Absorption at 500nm was taken and compared with a standard curve to calculate the cholesterol level. The standard curve was generated during each assay using cholesterol standards supplied with the Infinity reagent.

**Atherosclerotic lesion staining.** To examine atherosclerotic lesion formation and regression in the aorta, mice were perfused with saline solution for 3min, and then 4% paraformaldehyde solution for 6min. Abdominal organs and lungs were eviscerated. Under dissecting microscope, abdominal aorta was teased out, including the proximal part of femoral bifurcations. Working upwards, the following parts were dissected out, the descending aorta, the arch, and the ascending thoracic aorta including the three major branches (brachiocephalic, left carotid and subclavian). Then the entire artery tree from the aortic valve to bifurcations along with the attached heart was removed, and soaked in 4% paraformaldehyde solution for 24 hours at 4^o^C. For storage the artery tree was transferred to PBS with fungizone (Invitrogen). Aorta was dissected out, cut open along the inner side of the arch all the way down to the bifurcations. To flatten the arch region, an additional cut was made along the outer ridge of the arch, and the entire aorta was pinned down onto a block of black wax with 0.1mm minutein pins (Fine Science Tools, Foster City, CA). The flattened aorta was stained with the Sudan IV stain (0.5% Sudan IV in 35% ethanol/50% acetone/15% H2O) for 6min and de-stained with 80% ethanol for 5min to visualize the lesions. Photographs of the aortas were taken under dissecting microscope and used to quantify the lesion areas using imaging software.

**Statistical analysis.** To examine the statistical significance of ß-gal inducibility, Wilcoxon rank-sum test was used at an alpha level of 0.05. For the analysis of basal levels or induced levels of ß-gal, both Kruskal-Wallis rank sum test and pairwise Wilcoxon rank-sum tests were used with the criterion for statistical significance based on a “modified Bonferroni” approach. Thus pairwise Wilcoxon rank-sum tests were done each at an alpha level of 0.017 for each comparison. This will control overall type I error rate for each group of test at 0.05. Cholesterol levels were analyzed with 2-tailed Student’s t-test at an alpha level of 0.05. Comparison of atherosclerotic lesion areas was done using one-way ANOVA followed by Neuman-Keul's post hoc test at an alpha level of 0.05.

**Figure S1. Cre-mediated introduction of the insulator sequence into the lacZ gene at the predetermined loci.** (**A**) Vector structure for the introduction of the insulator sequence. This vector was used as a “TIGRE-targeting vector” shown in Figure 2C. (**B**) Predicted genomic structures. Parental clones (top) are the three ES clones T1, T2 and T3 shown in Figure 3B. Delta lacZ clones (middle) were generated by transient expression of Cre recombinase in parental clones followed by recombination between two loxP sites as shown in Figure 2B. Integrant clones (bottom) were generated by cotransfection of pGInZ (**A**) and Cre expression vector into delta lacZ clones as shown in Figure 2C. As a result, two copies of insulator sequences were introduced at both ends of the TRE-lacZ unit. ins, insulator. Other abbreviations are as described in Figure 2. (**C**) Analysis of Cre-mediated excision events by southern blot with KpnI digestion and probe neo. T1dZ, T2dZ, and T3dZ are delta lacZ clones generated by Cre-mediated excision from the parental ES clones T1, T2 and T3, respectively. (**D**) Analysis of Cre-mediated integration events by southern blot with EcoRV and probe TRE. T1inZ1-9, T2inZ1-9 and T3inZ1-3 are integrant clones created by Cre-mediated integration of pGInZ vector into the loxP site of the delta lacZ clones T1dZ, T2dZ and T3dZ, respectively. Unpredicted bands (indicated by arrowheads) were observed in four clones and they were not used for further analysis. (**E**) Analysis of Cre-mediated integration events by southern blot with KpnI and probe neo. Three clones which showed expected band in (**D**) were chosen from each integration site and analyzed together with corresponding delta lacZ clones. WT, wild type ES cells.

**Figure S2. Cre-mediated introduction of the luciferase gene without or with the insulator sequence into the predetermined loci.** (**A**) Vector structures for the introduction of the luciferase gene. pGTluc and pGIluc were used as a “TIGRE-targeting vector” shown in Figure 2C to introduce the luciferase gene without or with the insulator, respectively. (**B**) Predicted genomic structures after integration of pGTluc (top) and pGIluc (bottom) into the predetermined loci T1, T2 and T3. luc, luciferase. Other abbreviations are as described in Figure 2 and Figure S1. (**C**) Analysis of Cre-mediated integration events by southern blot with KpnI and probe neo. T1L1-3, T2L1-3 and T3L1-3 are integrant clones without the insulator generated by Cre-mediated integration of pGTluc vector into the loxP site of the delta lacZ clones T1dZ, T2dZ and T3dZ respectively (see Fig. S1). T1inL1-3, T2inL1-3 and T3inL1-3 are integrant clones with the insulator generated by Cre-mediated integration of pGIluc vector into the loxP site of the T1dZ, T2dZ and T3dZ, respectively. (**D**) Analysis of Cre-mediated integration events by southern blot with EcoRV and probe TRE. Unpredicted bands (indicated by arrowheads) were observed in two clones and they were not used for further analysis.

**Figure S3. Effect of Transrepressor on basal activity.** Using the tetracycline-dependent transrepressor is another way to reduce basal activity [5,17,18]. We tested the effect of transrepressor using the same ES clones introduced with insulator sequence. Although the effect on basal activity was similar between the two systems, our system is simpler because no additional protein expression (i.e. the transrepressor) is required. (**A**) Retroviral vector for the expression of the tetracycline-controlled transrepressor (tTR). P, PGK promoter. (**B**) Basal ß-gal activity after transduction with tTR. The same ES clones which was tested for insulator effect in Figures 3d and 3e were infected with the tTR retroviral vector, cultured in the presence of puromycin for 2 weeks, and basal ß-gal activities were measured from the mass culture containing more than 500 independent ES cell clones. Basal ß-gal activities were similar to those with the insulator shown in Figures 3D and 3E. Note that values are shown in logarithmic scale.

## Supplementary References

1. Bonnerot C, Rocancourt D, Briand P, Grimber G, Nicolas JF (1987) A beta-galactosidase hybrid protein targeted to nuclei as a marker for developmental studies. Proc Natl Acad Sci U S A 84: 6795-6799.

2. Soriano P, Montgomery C, Geske R, Bradley A (1991) Targeted disruption of the c-src proto-oncogene leads to osteopetrosis in mice. Cell 64: 693-702.

3. Soriano P, Friedrich G, Lawinger P (1991) Promoter interactions in retrovirus vectors introduced into fibroblasts and embryonic stem cells. J Virol 65: 2314-2319.

4. Chung JH, Whiteley M, Felsenfeld G (1993) A 5' element of the chicken beta-globin domain serves as an insulator in human erythroid cells and protects against position effect in Drosophila. Cell 74: 505-514.

5. Forster K, Helbl V, Lederer T, Urlinger S, Wittenburg N, et al. (1999) Tetracycline-inducible expression systems with reduced basal activity in mammalian cells. Nucleic Acids Res 27: 708-710.

6. Stauber RH, Horie K, Carney P, Hudson EA, Tarasova NI, et al. (1998) Development and applications of enhanced green fluorescent protein mutants. Biotechniques 24: 462-466, 468-471.

7. Soneoka Y, Cannon PM, Ramsdale EE, Griffiths JC, Romano G, et al. (1995) A transient three-plasmid expression system for the production of high titer retroviral vectors. Nucleic Acids Res 23: 628-633.

8. Yee JK, Miyanohara A, LaPorte P, Bouic K, Burns JC, et al. (1994) A general method for the generation of high-titer, pantropic retroviral vectors: highly efficient infection of primary hepatocytes. Proc Natl Acad Sci U S A 91: 9564-9568.

9. Swiatek PJ, Gridley T (1993) Perinatal lethality and defects in hindbrain development in mice homozygous for a targeted mutation of the zinc finger gene Krox20. Genes Dev 7: 2071-2084.

10. Tessarollo L (2001) Manipulating mouse embryonic stem cells. Methods Mol Biol 158: 47-63.

11. Gu H, Zou YR, Rajewsky K (1993) Independent control of immunoglobulin switch recombination at individual switch regions evidenced through Cre-loxP-mediated gene targeting. Cell 73: 1155-1164.

12. Sauer B, Henderson N (1990) Targeted insertion of exogenous DNA into the eukaryotic genome by the Cre recombinase. New Biol 2: 441-449.

13. Tyagi S, Kramer FR (1996) Molecular beacons: probes that fluoresce upon hybridization. Nat Biotechnol 14: 303-308.

14. Resnitzky D, Gossen M, Bujard H, Reed SI (1994) Acceleration of the G1/S phase transition by expression of cyclins D1 and E with an inducible system. Mol Cell Biol 14: 1669-1679.

15. Shaper NL, Harduin-Lepers A, Shaper JH (1994) Male germ cell expression of murine beta 4-galactosyltransferase. A 796-base pair genomic region, containing two cAMP-responsive element (CRE)-like elements, mediates male germ cell-specific expression in transgenic mice. J Biol Chem 269: 25165-25171.

16. Young DC, Kingsley SD, Ryan KA, Dutko FJ (1993) Selective inactivation of eukaryotic beta-galactosidase in assays for inhibitors of HIV-1 TAT using bacterial beta-galactosidase as a reporter enzyme. Anal Biochem 215: 24-30.

17. Freundlieb S, Schirra-Muller C, Bujard H (1999) A tetracycline controlled activation/repression system with increased potential for gene transfer into mammalian cells. J Gene Med 1: 4-12.

18. Rossi FM, Guicherit OM, Spicher A, Kringstein AM, Fatyol K, et al. (1998) Tetracycline-regulatable factors with distinct dimerization domains allow reversible growth inhibition by p16. Nat Genet 20: 389-393.
